# AccA from *Neisseria gonorrhoeae* provides a new framework for understanding periplasmic copper metallochaperones

**DOI:** 10.1039/d5sc08738d

**Published:** 2026-03-12

**Authors:** Samantha Firth, William Earl, Denis Thaqi, YoungJin Hong, Charlotte O'Hern, Gemma Luscombe, Dalton Heng Yong Ngu, Zhenyao Luo, Chacko Jobichen, Bostjan Kobe, Alastair McEwan, Karrera Djoko

**Affiliations:** a Department of Biosciences, Durham University Durham DH1 3LE UK samantha.j.firth@durham.ac.uk karrera.djoko@durham.ac.uk; b School of Chemistry and Molecular Biosciences, The University of Queensland St Lucia QLD 4072 Australia; c Institute for Molecular Bioscience, The University of Queensland St Lucia QLD 4072 Australia; d Australian Infectious Diseases Research Centre, The University of Queensland St Lucia QLD 4072 Australia

## Abstract

Many bacteria use copper (Cu) to drive key redox reactions and energy metabolism, and they often rely on metallochaperones to deliver Cu to Cu-dependent enzymes. However, why delivery by metallochaperones is needed, and why Cu cannot transfer directly from cellular pools to the target enzymes, is not well understood. Here, we show that the PCu_A_C-family metallochaperone AccA from the periplasm of *Neisseria gonorrhoeae* delivers Cu to the Cu-dependent nitrite reductase AniA, enabling growth and nitrite respiration in O_2_-limiting conditions. Although purified AccA binds both Cu(i) and Cu(ii) ions, only the Cu(i)-binding site is essential for activating AniA in *N. gonorrhoeae* cells. Unexpectedly, the Cu(i)-binding affinity of AniA is >50 times weaker than that of AccA, suggesting that Cu delivery occurs against a favourable affinity gradient. We propose that AccA is needed because AniA cannot compete with the periplasmic milieu for binding Cu, providing a new framework to understand why some Cu-dependent enzymes need metallochaperones to deliver Cu.

## Abstract 2

The AccA metallochaperone from *Neisseria gonorrhoeae* delivers Cu(i) against a favourable thermodynamic gradient to the Cu-dependent nitrite reductase AniA.

## Graphical abstract

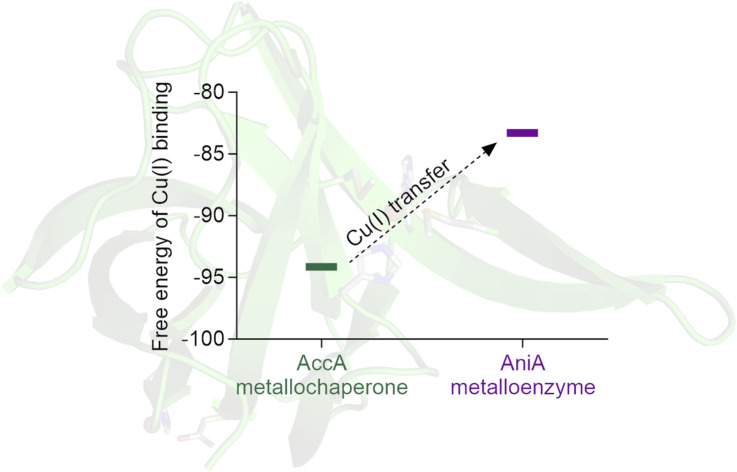

## Introduction

Copper (Cu) is essential for bacterial physiology, particularly as a redox catalyst in respiration and nutrient acquisition.^1–10^ However, ensuring that Cu reaches the right proteins is a major challenge for cells. Proteins follow the Irving–Williams series and universally prefer to bind Cu than other metals. In the cytoplasm, widespread mis-metalation by Cu does not occur because the cytoplasmic milieu buffers metal availabilities in the inverse order, with Cu being very low.^11^ From this buffer, Cu travels through a relay of ligand exchange steps following a favourable thermodynamic gradient,^12^*i.e.*, from less stable (weaker binding) to more stable (stronger binding) sites, until it reaches its final destinations, namely the Cu-dependent enzymes (cuproenzymes) that require Cu for activity.

Bacterial cuproenzymes are localised outside the cytoplasm, *i.e.*, in the membrane or periplasm of Gram-negative bacteria or in the membrane or on the surface of Gram-positive bacteria. These compartments are essentially exposed to the external environment, and it is unclear whether an effective Cu buffer operates here. Cu insertion into many of these extracytoplasmic cuproenzymes often requires the assistance of extracytoplasmic Cu-binding metallochaperones.^13–16^ The best understood Cu metallochaperones act under conditions of Cu surplus, scavenging the excess Cu and directing it towards detoxification or efflux pathways.^17^ Others function during Cu limitation and help ensure that cuproenzymes receive nutrient Cu,^15,18^ but how they do so is not well understood.

The PCu_A_C (periplasmic Cu_A_ chaperones) family of extra-cytoplasmic Cu metallochaperones assists in the assembly of the dinuclear, mixed-valence Cu_A_ centre in cytochrome *c* oxidases (Ccos)^19–24^ in many bacteria. These metallochaperones share a characteristic cupredoxin-like fold and a conserved HX_*n*_MX_21/22_HXM motif (here “primary site”; Fig. S1) that binds one Cu(i) ion. Some PCu_A_Cs also have a short C-terminal extension that is rich in His and Met (here “tail”; Fig. S1). In PCu_A_C from *Bradyrhizobium diazoefficiens* (Bd-PCu_A_C), the tail binds Cu(ii).^21^ This tail is essential for assembling the Cu_A_ centre in Bd-Cco, at least in experiments with purified proteins.^21^ By contrast, this tail is absent in PCu_A_C from *Thermus thermophilus* (Tt-PCu_A_C).^20^ Experiments with purified Tt-PCu_A_C protein showed that the conserved primary site is sufficient for correct assembly of the Cu_A_ centre in Tt-Cco.^20^ A separate group of PCu_A_Cs from methanotrophs lacks the primary site motif entirely and instead has a HX_10_H His-brace in a different position.^25^ These PCu_A_Cs are likely important for assembling the copper centres in particulate methane monooxygenases, although this proposal awaits experimental evidence.

The Gram-negative bacteria *Neisseria meningitidis* and *Neisseria gonorrhoeae* do not have Ccos with Cu_A_ centres. Nonetheless, they have a PCu_A_C homologue named AccA (Fig. S1), whose likely role is to insert Cu into a Cu-dependent nitrite reductase (NirK) named AniA.^26^ Like other NirKs, the mature AniA enzyme is a homotrimer with mononuclear Type 1 Cu (T1Cu) and Type 2 Cu (T2Cu) centres in each active site.^27–29^ Mutant strains of *N. meningitidis* and *N. gonorrhoeae* missing the intact *accA* gene produced the AniA polypeptide but failed to grow using nitrite as the terminal electron acceptor for respiration.^26^ Growth and nitrite respiration were restored by adding Cu to the cultures, suggesting that, without AccA, AniA is produced in its Cu-free (*apo*-) form. Purified AccA from *N. meningitidis* binds two Cu ions: one Cu(i), presumably at the conserved primary site, and one Cu(ii), presumably at the tail (Fig. S1).^26^ This protein interacts with purified AniA with and without Cu,^26^ but Cu transfer between them has yet to be demonstrated. Unlike previously studied PCu_A_Cs, AccA has additional His and Met residues near the primary site (here “track”; Fig. S1). Which of the primary, tail, or track sites is important for metalating AniA has not been identified.

Why AccA is needed but not when cells are cultured with supplemental Cu^26^ is puzzling. This conditional essentiality was also seen in studies of other PCu_A_Cs^21–24^ but cannot be explained by current thermodynamic models of Cu delivery.^12,15,30^ If Cu can transfer from a weaker site in the periplasm to the metallochaperone, and subsequently from the metallochaperone to the target cuproenzyme, why can Cu not transfer directly to the cuproenzyme? Why does adding Cu overcome the loss of the metallochaperone? Here we show that AccA from *N. gonorrhoeae* directly inserts Cu into AniA, and thus establish a new role for PCu_A_Cs in the assembly of NirK enzymes. By integrating culture phenotypes of *N. gonorrhoeae* mutant strains with Cu binding affinities of purified AccA and AniA proteins, we propose a modified thermodynamic model for Cu delivery by AccA (or indeed other PCu_A_Cs) that resolves previously puzzling aspects of its function. The ideas outlined here potentially shape how we understand the PCu_A_C (and other) family of metallochaperones.

## Results and discussion

### AccA binds to two Cu ions – one Cu(i) and one Cu(ii)

To examine the Cu-binding properties of AccA, we overexpressed it in *Escherichia coli*. Our overexpression construct used the native Sec signal sequence from *N. gonorrhoeae.* As expected from computational prediction,^31^ this construct was processed by *E. coli* to generate Ala20 as the N-terminal residue (Fig. S1 and Table S5). Incubating purified *apo*-wild-type (WT)-AccA with excess Cu_aq_^2+^ followed by desalting led to 1.9 ± 0.1 eq. of bound Cu(ii) in the protein. Adding ascorbate during the incubation step reduced one of these bound Cu(ii) ions, yielding 0.93 ± 0.03 eq. of Cu(i) and 1.0 ± 0.1 eq. of Cu(ii). The bound Cu(i) ion was air-stable for many hours following removal of ascorbate. These stoichiometries support the previous proposal^26^ that AccA has two Cu-binding sites, one each for Cu(i) and Cu(ii).

### The conserved HX_*n*_MX_21/22_HXM motif binds Cu(i) with sub-femtomolar affinity

**Fig. 1:**
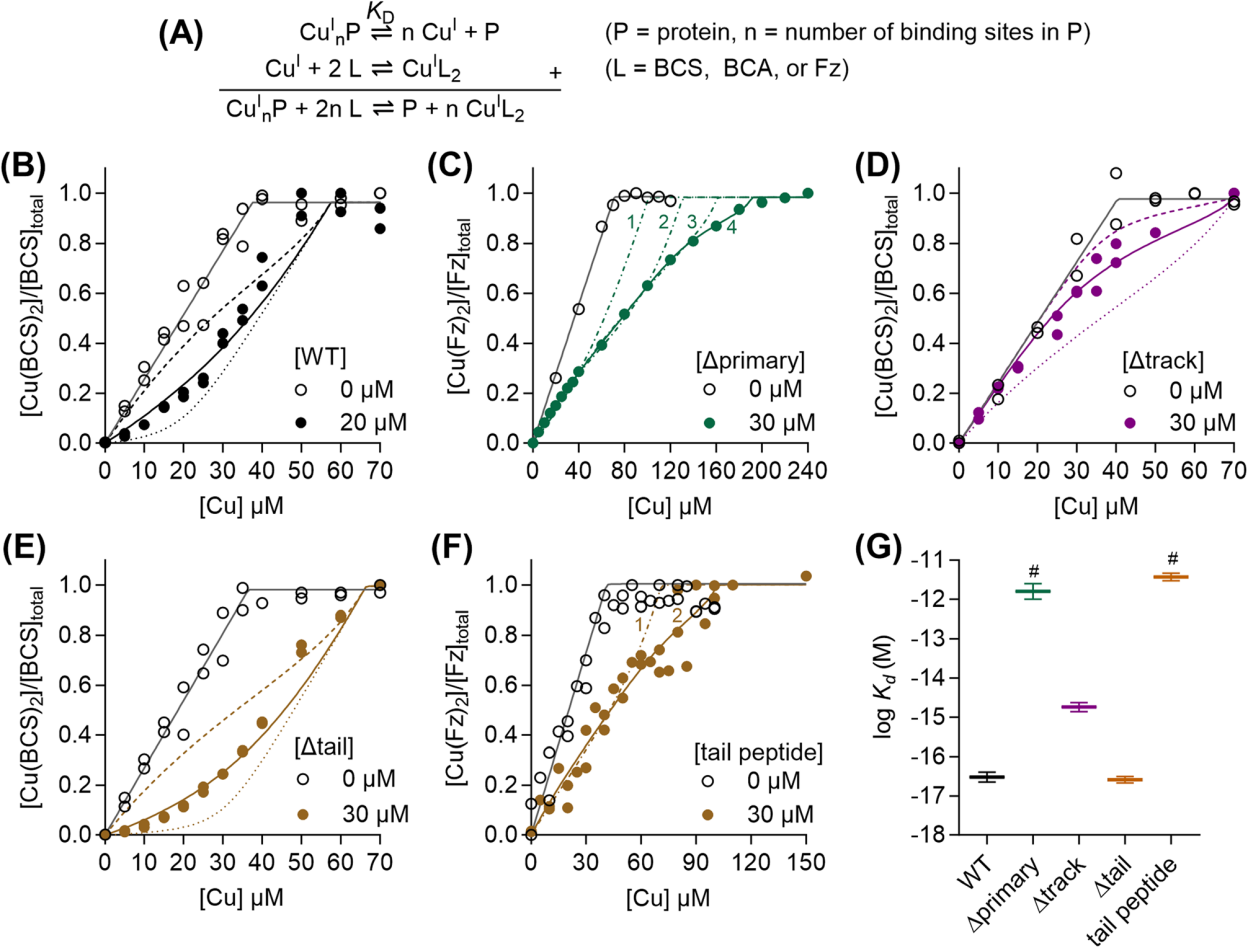
Cu(i)-binding affinity of AccA. (A) Equations describing the ligand equilibrium competition approach. Competition curves between: (B) WT-AccA (0 or 20 µM) and BCS (75 µM), (C) Δprimary-AccA (0 or 30 µM) and Fz (142 µM), (D) Δtrack-AccA (0 or 30 µM) and BCS (81 µM), (E) Δtail-AccA (0 or 30 µM) and BCS (73 µM), and (F) the synthetic tail peptide (0 or 30 µM) and Fz (82 µM). Individual data points are shown. Competition curve fits (coloured solid lines) produced the log *K*_D_ values shown in panel (G). Control curve fits (black solid lines), simulated fits for 10× lower (dotted lines) or 10× higher (dashed lines) *K*_D_ values, and simulated fits for different values for *n* (1, 2, 3, or 4; dot-dashed lines) are also shown. (G) Summary of Cu(i)-binding affinities (horizontal lines). Error bars represent ± SEM. ^#^Values represent lower limits only.

To determine the Cu(i)-binding affinity of WT-AccA, it was competed with the colorimetric Cu(i)-binding probes Fz, BCA, or BCS (Table 1) in the presence of ascorbate (Fig. 1A).^32^ WT-AccA outcompeted Fz and BCA but competed effectively with BCS (Fig. 1B), allowing us to estimate a log *K*_D_ of −16.5 ± 0.1 M for this protein (Fig. 1G and Table 1). This sub-femtomolar affinity is orders of magnitude tighter than that for Tt-PCu_A_C (log *K*_D_ = −12.7 M, revisited in Fig. 7A)^20^ but comparable to those for Bd-PCu_A_C (log *K*_D_ < −16 M)^22^ and the PCu_A_C from *Streptomyces lividans* (Sl-PCu_A_C; log *K*_D_ = −15.7 M).^19^

**Table 1:** Cu(i) and Cu(ii)-binding affinities for: (A) proteins determined in this work and (B) probes used in this work.

	log *K*_D_ (M)	
	Cu(i)	Cu(ii)
**(A) Proteins**		
WT AccA	−16.5 ± 0.1	−12.4 ± 0.1
H69A-AccA	−13.6 ± 0.1	
M80A-AccA	−13.9 ± 0.1	
H103A-AccA	−13.9 ± 0.1	
M105A-AccA	−13.5 ± 0.1	
ΔPrimary-AccA	−11.8 ± 0.2	−11.8 ± 0.0
ΔTrack-AccA	−14.7 ± 0.1	−12.2 ± 0.1
ΔTail-AccA	−16.6 ± 0.1	−9.5 ± 0.1
Tail peptide	−11.4 ± 0.1	−11.6 ± 0.1
Tt-PCuAC	−16.2 ± 0.1	
AniA	−14.7 ± 0.1	−12.4 ± 0.1

**(B) Probes** ^32^		
Fz^a^	−15.1	
BCA^a^	−17.2	
BCS^a^	−19.8	
DP-3		−12.3
DP-2		−10.1

a Values shown are log 1/*β*_2_ (M^2^).

AccA variants missing a single residue from the conserved HX_*n*_MX_21/22_HXM motif (Fig. S1) competed effectively with BCA (Fig. S2A) but not BCS. Their estimated *K*_D_ values were in the sub-picomolar range and similar to one another (Fig. S2B and Table 1). The Δprimary-AccA variant missing all four residues competed with Fz (Fig. 1C) but not BCS or BCA. The competition curve was best fitted to a model with four Cu(i)-binding sites in the protein, the tightest of which has a picomolar affinity (Fig. 1C and Table 1). However, spectral changes in this experiment (Fig. S3) led us to suspect formation of ternary AccA-Cu(i)-Fz complexes. Although a meaningful affinity was not determined for this variant, it was >4 orders of magnitude weaker than that for WT-AccA (Fig. 1G and Table 1), establishing the role of the conserved motif as the main binding site for Cu(i).

AccA has additional, non-conserved Met and His residues: three near the primary site (track) and eight at the C-terminus (tail) (Fig. S1). ΔTrack-AccA competed weakly with BCS (Fig. 1D) but outcompeted BCA, indicating a supra-femtomolar Cu(i)-binding affinity for this variant (Fig. 1G and Table 1). The Δtail-AccA variant resembled WT-AccA and competed with BCS (Fig. 1E). By contrast, a synthetic 16-mer peptide corresponding to the tail alone (Fig. S1) resembled the Δprimary-AccA variant – it did not compete with BCS or BCA and it formed a ternary complex with Fz. A line of best fit was obtained by modelling two Cu(i)-binding sites in this peptide, both with near-picomolar affinities (Fig. 1F and Table 1). Therefore, neither the track nor the His/Met-rich tail is the main binding site for Cu(i).

### The C-terminal His/Met-rich extension binds Cu(ii) with picomolar affinity

**Fig. 2:**
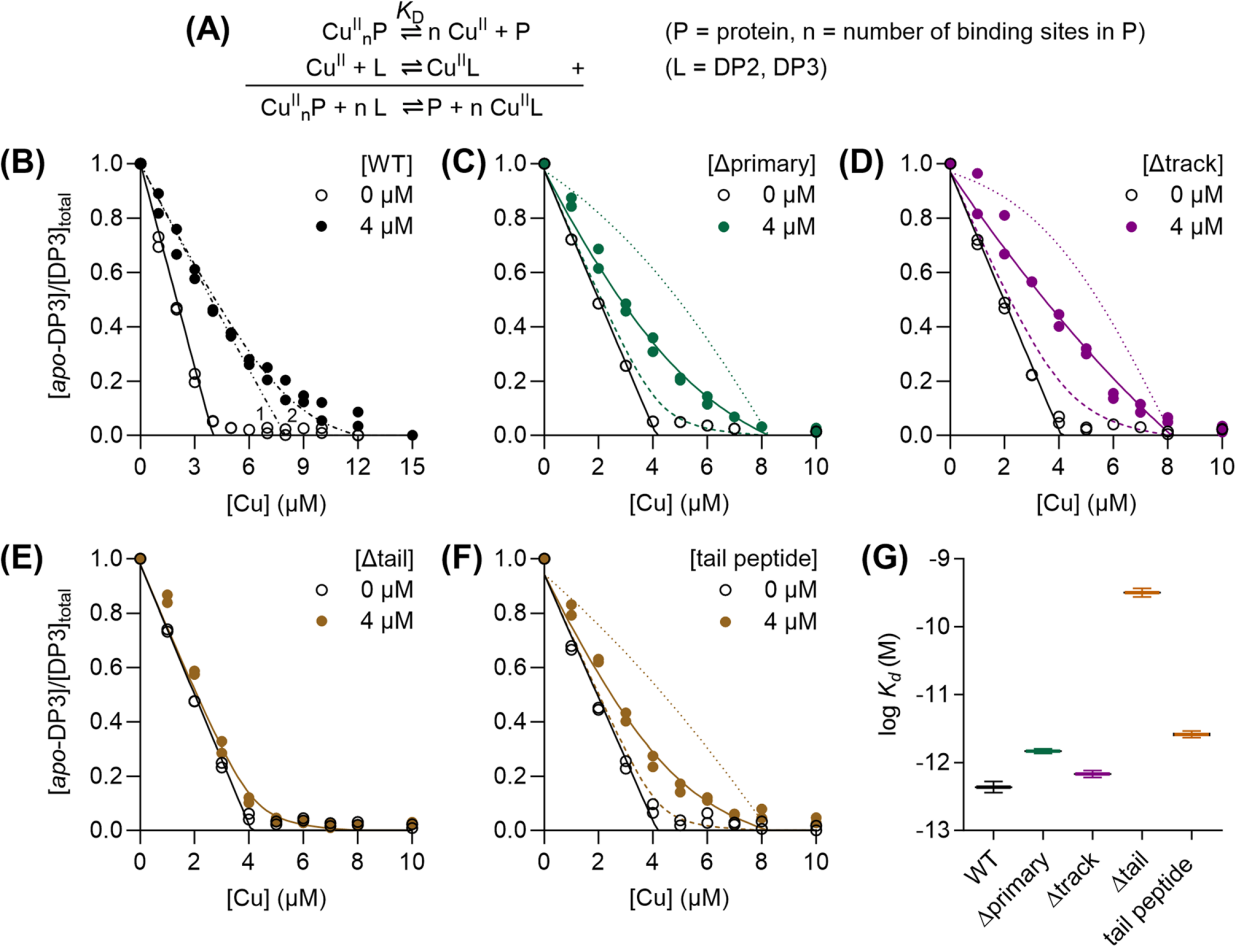
Cu(ii)-binding affinity of AccA. (A) Equations describing the ligand equilibrium competition approach. Competition curves between DP3 (4 µM) and 0 or 4 µM each of: (B) WT-AccA, (C) Δprimary-AccA, (D) Δtrack-AccA, (E) Δtail-AccA, and (F) the synthetic tail peptide. Individual data points are shown. Competition curve fits (coloured solid lines) produced the log *K*_D_ values shown in panel (G). Control curve fits (black solid lines), simulated fits for 10× lower (dotted lines) or 10× higher (dashed lines) *K*_D_ values, and simulated fits for different values for *n* (1 or 2; dot-dashed lines) are also shown. (G) Summary of Cu(ii)-binding affinities (horizontal lines). Error bars represent ± SEM.

To determine the Cu(ii)-binding affinities of AccA proteins, each was competed with the fluorometric, Cu(ii)-binding dansyl peptide DP3 (Table 1)^33^ without ascorbate (Fig. 2A and S4A). WT-AccA competed effectively with this probe (Fig. 2B). The competition curve was best modelled with two Cu(ii)-binding sites in AccA, the tightest of which binds with a log *K*_D_ of −12.4 ± 0.1 M (Fig. 2B, G, and Table 1). Mutating all primary or all track residues eliminated only the weaker site (Fig. 2C and D) while deleting the entire tail abolished both sites (Fig. 2E). When competed with the lower affinity peptide DP2 (Table 1),^33^ the Δtail-AccA variant showed only one Cu(ii)-binding site with a weak, nanomolar affinity (Fig. S4B, 2G, and Table 1). Finally, the synthetic tail peptide showed only one, picomolar affinity site that competed with DP3 (Fig. 2F, G, and Table 1) and a second, nanomolar affinity site that competed with DP2 (Fig. S4B and Table 1). Altogether, these data indicate that the C-terminal His/Met-rich tail provides the main ligands for binding Cu(ii).

### X-ray crystal structure of Cu(i)- and Cu(ii)-loaded AccA

**Fig. 3:**
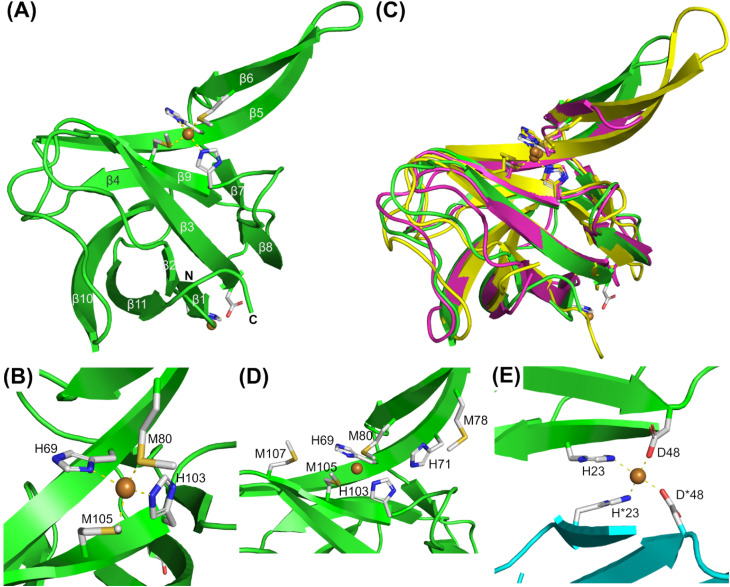
X-ray crystal structure of Cu(i)- and Cu(ii)-loaded AccA. (A) Cartoon representation of the 2.9 Å resolution structure of Cu(i) and Cu(ii)-loaded WT-AccA (green; residues 20–141 could be modelled (*cf.* Fig. S1A); PDB 9YAH). (B) Zoomed-in view of the primary site residues. (C) Structure of AccA (green) superimposed onto the X-ray crystal structure of Cu(i)-Sl-PCu_A_C (pink; PDB 3ZJA^19^) and the NMR solution structure of Cu(i)-Tt-PCu_A_C (yellow; PDB 2K70 ^20^). (D) Arrangement of the track residues. (E) Zoomed-in view of the second Cu ion. This Cu ion is coordinated by H23 and D48 from the monomer in the crystallographic asymmetric unit (green) and its symmetry mate (cyan).

Although *apo*- and Cu-loaded AccA proteins were prepared from the same batch of purified AccA, using the same buffers, and screened using the same conditions, only Cu-loaded AccA crystals were obtained. The crystal structure of this Cu-bound AccA was determined using single-wavelength anomalous diffraction phasing based on the copper ion (Table S7). The crystals contained one protein molecule in the crystallographic asymmetric unit. The structure features a cupredoxin-like barrel core composed of 11 twisted, antiparallel β-strands, with strands β5 and β6 forming a “finger-like” β hairpin that protrudes from the barrel (Fig. 3A). The conserved His and Met residues from the primary site coordinate a single Cu ion in a near-tetrahedral coordination geometry (Fig. 3B), consistent with Cu(i).

The cupredoxin barrel, the positions of primary site ligands, and the coordination geometry of the Cu ion in AccA are essentially identical to those of other structurally characterised Cu(i)-PCu_A_C proteins (Fig. 3C). By contrast, the track (Fig. 3D) is not conserved. Although track residues do not directly coordinate the Cu ion in our crystal structure, their removal weakened the Cu(i)-binding affinity of AccA (Fig. 1G). This finding suggests that track residues help stabilise the bound Cu(i) ion in solution. There was insufficient electron density in the structure to model the His/Met-rich tail. Nevertheless, the terminal modelled residues (Lys138 to Pro141) suggested that this tail projects outward from the cupredoxin barrel towards the solvent (Fig. 3A). The tail is likely disordered and flexible, as the circular dichroism spectrum of the synthetic tail peptide showed little β-sheet or α-helix character, irrespective of the presence of Cu (Fig. S5).

The AccA structure contains a second Cu ion, coordinated by His23 and Asp48 from the monomer in the asymmetric unit and the same two residues from the symmetry-related molecule (Fig. 3E). This arrangement creates a Cu-bridged dimer with a distorted square planar coordination geometry around the Cu centre, consistent with Cu(ii). However, addition of Cu(i) and Cu(ii) into *apo*-AccA did not promote dimer formation in solution (Fig. S6). In addition, Cu-binding measurements firmly established the tail, and not the cupredoxin barrel, as the main, picomolar affinity site for Cu(ii) (Fig. 2G). Therefore, this second Cu site in the AccA crystal is likely a weak, non-specific site that is not relevant biologically.

### The conserved HX_*n*_MX_21/22_HXM motif but not the C-terminal His/Met-rich extension is essential in *N. gonorrhoeae*

**Fig. 4:**
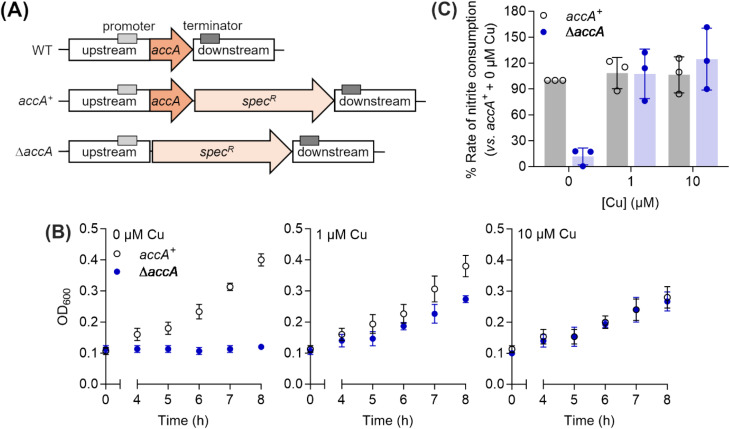
Culture phenotypes of the *N. gonorrhoeae* Δ*accA* mutant strain. (A) Genetic constructs used in this study. (B) Growth of *N. gonorrhoeae accA*^*+*^ and Δ*accA* mutant strains with 0, 1, or 10 µM of added Cu. Data points and error bars represent the means (*N* = 3) and ±SD, respectively. (C) Rates of nitrite consumption by whole *N. gonorrhoeae accA*^*+*^ and Δ*accA* mutant cells from the 8-hour time-point in panel (B). Data points from individual replicates (*N* = 3) are shown. Columns and error bars represent means and ± SD, respectively.

To assess the functional importance of the different Cu-binding sites in AccA, we created mutant strains of *N. gonorrhoeae* in which these sites were mutated to Ala or deleted entirely. These mutations were introduced into the original *accA* locus to preserve its native transcriptional control. As the selection marker, a promoterless spectinomycin-resistance cassette (*spec*^*R*^) was inserted downstream of the *accA* coding sequence but upstream of its native terminator (Fig. 4A). This cassette did not affect transcription of adjacent genes, bacterial growth, or nitrite consumption by whole cells (Fig. S7). Therefore, a strain carrying wild-type *accA* gene with *spec*^*R*^ (Fig. 4A) was used as the isogenic *accA*^*+*^ control in all experiments.

**Fig. 5:**
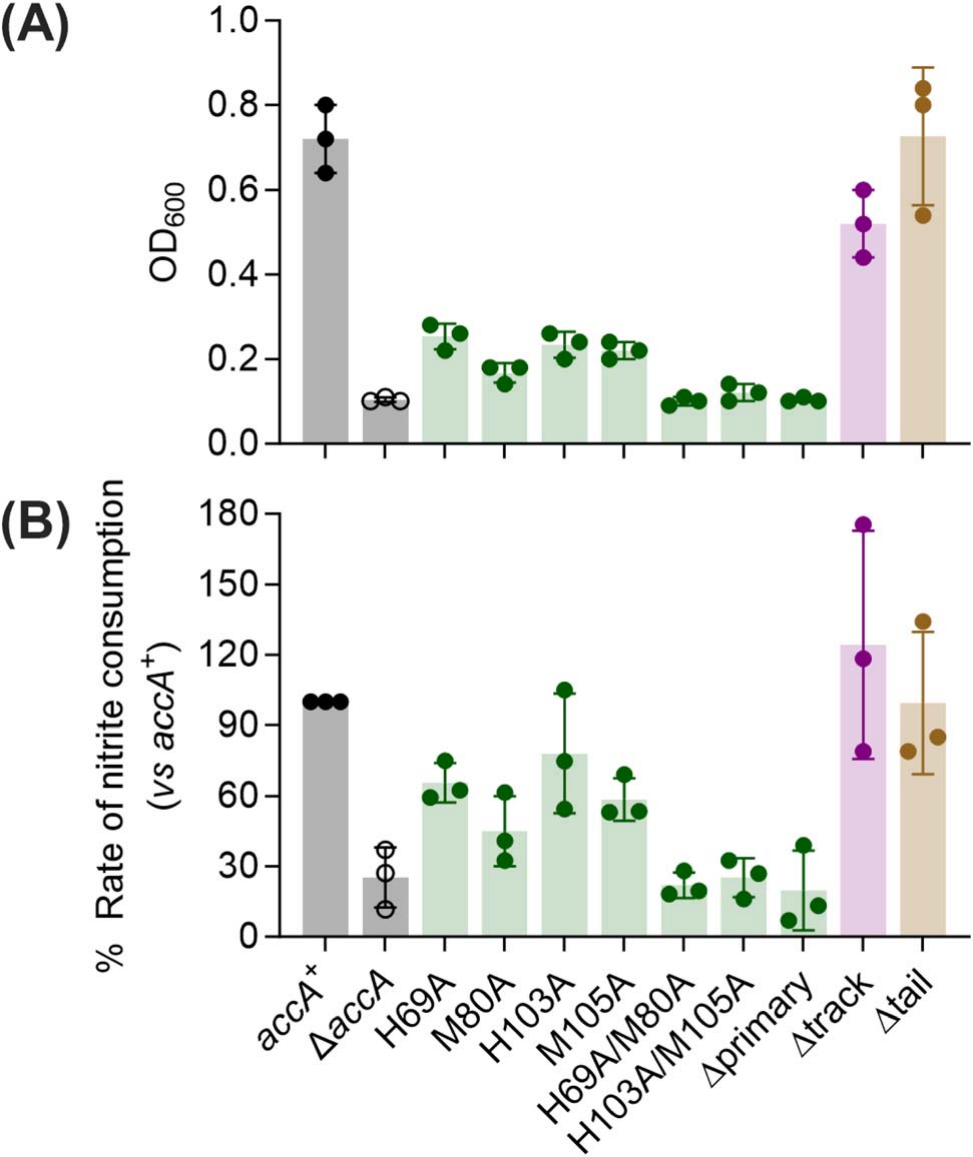
Culture phenotypes of *N. gonorrhoeae accA* site-directed mutant strains. (A) Final culture densities after growth for 8 h without added Cu. (B) Rates of nitrite consumption by whole cells from panel (A). In both panels, data from individual replicates (*N* = 3) are shown. Columns and error bars represent means and ± SD, respectively.

As reported previously,^26^ a Δ*accA* strain missing the *accA* gene did not grow or consume nitrite unless Cu was added to the cultures (Fig. 4B and C). Mutating any one of the primary site residues to Ala partially reduced growth and nitrite consumption (Fig. 5, S8A–D, and S9) but mutating more than one residue produced a Δ*accA*-like phenotype (Fig. 5, S8E–G, and S9). Deleting the entire C-terminal tail had no effect (Fig. 5, S10A, and C). Mutating all track residues to Ala slightly reduced growth but this effect became more pronounced in the presence of the Cu(i) chelator BCS (Fig. 5, S10B, D). In these Cu-limiting conditions, the Δtrack-*accA* mutant strain did not grow and only partially consumed nitrite (Fig. S10B and D). All defects in growth and nitrite consumption were rescued by adding Cu to the cultures (Fig. S8–S10). However, we note that adding 10 µM of Cu mildly suppressed growth of the *accA*^*+*^ strain compared with the untreated control (Fig. 4B and S8), suggesting that this concentration of Cu is mildly toxic to *N. gonorrhoeae*.

Overall, these culture phenotypes mirrored the Cu(i)-binding affinities of AccA (Fig. 1G): strains expressing weaker-binding AccA proteins grew and consumed nitrite less efficiently. Therefore, we concluded that the conserved Cu(i)-binding motif is essential for the function of AccA in *N. gonorrhoeae*. While not essential, the track may play a role, especially under Cu-limiting conditions. By contrast, the Cu(ii)-binding, C-terminal His/Met-rich tail is dispensable.

### AccA reconstitutes the T1Cu centre in AniA

**Fig. 6:**
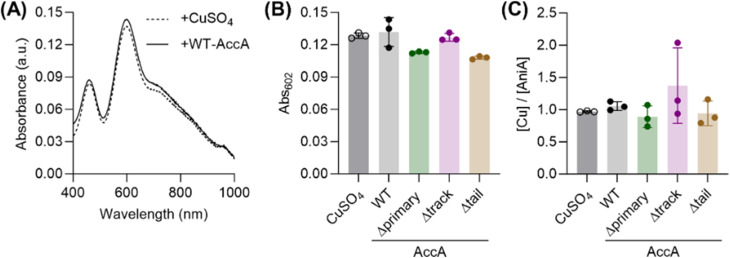
Transfer of Cu from AccA to AniA. (A) Solution spectra of AniA after incubation with 2–3 eq. of CuSO_4_ or Cu(i)Cu(ii)-loaded form of WT-AccA. Representative traces from *N* = 3 experiments are shown. (B) Solution absorbance intensities of AniA at 602 nm after ∼1 h of incubation with 2–3 eq. of CuSO_4_ or Cu(i)Cu(ii)-loaded forms of AccA proteins. Data points from individual replicates (*N* = 3) are shown. Columns and error bars represent means and ±SD, respectively. (C) Cu-binding stoichiometries of AniA from the experiment in panel (B), determined following separation of AniA and AccA. Data points from individual replicates (*N* = 3) are shown. Columns and error bars represent means and ±SD, respectively.

Purified AccA and AniA proteins were previously shown to interact.^26^ To test whether AccA acts as a metallochaperone for AniA, we examined transfer of Cu from purified AccA to the soluble domain of AniA. First, we overexpressed the soluble domain of AniA in *E. coli* without its N-terminal signal sequence, N-terminal lipidated sequence, and C-terminal glycosylated domain, as described previously.^27,28^ Next, adding Cu_aq_^2+^ to purified *apo*-AniA produced optical features characteristic of a T1Cu centre (Fig. 6A and S11). The final spectrum showed the expected^27,28^ absorbances at 602 and 462 nm, and an *A*_462_/*A*_602_ ratio of 0.65 (Fig. 6A). The T2Cu centre has no optical features but incubation with excess Cu_aq_^2+^ followed by desalting produced a Cu:AniA stoichiometry of 1.9 (±0.1), consistent with both Cu centres being present. Therefore, we used the optical features of the T1Cu centre to monitor Cu transfer from AccA to AniA.

Adding the Cu(i)- and Cu(ii)-loaded form of WT-AccA (∼2.5 eq.) to *apo*-AniA also produced the expected T1Cu absorbances at 602 nm (Fig. 6A and B). These results indicate that AccA acts as a metallochaperone that inserts Cu into the T1Cu site in AniA. Following separation of the two proteins on an anion exchange column, only ∼1 eq. of Cu was found in AniA-containing fractions (Fig. 6C), suggesting that AccA does not insert Cu into the T2Cu site in AniA. However, the T2Cu centre in NirK proteins is known to dissociate during ion exchange chromatography.^34,35^ Indeed, elution of Cu_aq_^2+^-loaded AniA on the same column produced the same stoichiometry of ∼1 eq. of bound Cu (Fig. 6C). Therefore, whether AccA also inserts Cu into the T2Cu site in AniA awaits further investigation.

Like WT-AccA, the Cu(i)Cu(ii)-loaded forms of Δtrack-AccA and Δtail-AccA variants also reconstituted the T1Cu centre in AniA (Fig. 6B). Thus, it appears that neither the track nor the tail is essential for interacting with and/or transferring Cu to AniA, consistent with our finding that neither is essential for growth or nitrite consumption by *N. gonorrhoeae* (Fig. 5). Interestingly, Cu(i)Cu(ii)-loaded Δprimary-AccA also reconstituted the T1Cu signal (Fig. 6B), even though the corresponding *N. gonorrhoeae* Δprimary-*accA* cultures did not produce a functional AniA (Fig. 5). It must be noted that Δprimary-AccA can bind one Cu(i) ion with picomolar affinity (Fig. 1G), which was partially retained (0.3 ± 0.1 eq.) after desalting. Based on these results, we propose a simple model in which bound Cu in any site in any purified AccA variant can be transferred to purified AniA (examined further below).

### Tt-PCu_A_C, which has the conserved HX_*n*_MX_21/22_HXM motif but not the track or His/Met-rich C-terminal extension, does not functionally substitute for AccA

**Fig. 7:**
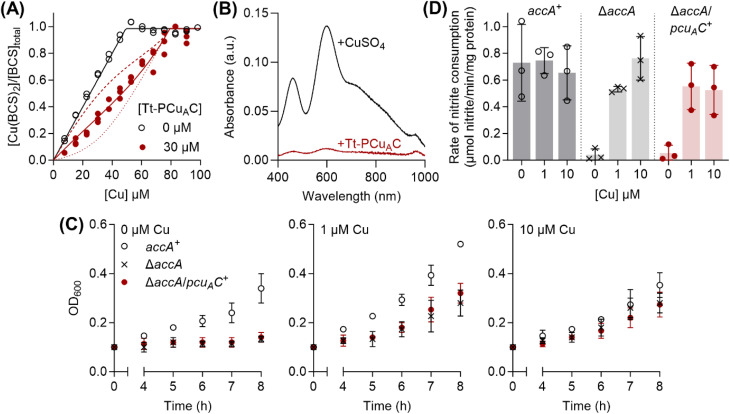
Tt-PCu_A_C does not functionally substitute for AccA. (A) Cu(i)-binding affinity of Tt-PCu_A_C. Competition curve between BCS (98.5 µM) and Tt-PCu_A_C (0 or 30 µM). The competition approach is shown in Fig. 1A. Individual data points are shown. Competition curve fits (red solid line) produced the log *K*_D_ value shown in the text. Control curve fit (black solid line) and simulated fits for 10× lower (dotted lines) or 10× higher (dashed lines) *K*_D_ values are also shown. (B) Solution spectra of AniA after incubation with 2–3 eq. of CuSO_4_ or Cu(i)-Tt-PCu_A_C. Representative traces from *N* = 3 experiments are shown. (C) Growth of *N. gonorrhoeae accA*^*+*^, Δ*accA*, and Δ*accA*/*pcu*_*A*_*C*^*+*^ mutant strains with 0, 1 or 10 µM of added Cu. Data points and error bars represent means (*N* = 3) and ±SD, respectively. (D) Rates of nitrite consumption by whole *accA*^*+*^, Δ*accA*, and Δ*accA*/*pcu*_*A*_*C*^*+*^ mutant cells from the 8-hour time-point in panel (C). Data from individual replicates (*N* = 3) are shown. Columns and error bars represent means and ±SD, respectively.

Given the essentiality of the conserved primary site of AccA, but not the track or tail, in *N. gonorrhoeae* cultures (Fig. 5), we were curious whether Tt-PCu_A_C, which lacks the track and tail, inserts Cu into AniA. We first confirmed that, as expected,^20^ incubating purified *apo*-Tt-PCu_A_C with excess Cu and ascorbate led to co-elution of the protein with only ∼1 eq. of Cu(i) from a desalting column. This protein competed effectively with BCS (Fig. 7A), yielding a log *K*_D_ of −16.2 ± 0.1 M. The previously reported value (log *K*_D_ = −12.7)^20^ is ∼3000-fold higher, likely because it was obtained using DTT as the competitor and a Cu(i)-binding affinity for DTT that was underestimated by ∼10 000-fold.^36^ More importantly, when measured using identical conditions, Tt-PCu_A_C (Fig. 7A) and AccA (Fig. 1G) showed comparable affinities for Cu(i).

Adding Cu(i)-loaded Tt-PCu_A_C to *apo*-AniA failed to produce the characteristic T1Cu optical signals (Fig. 7B). Because Cu(i)-AniA is optically silent,^28^ we also measured the amounts of Cu bound to AniA following separation of the two proteins on an anion exchange column. Only <0.1 eq. of Cu was detected in AniA-containing fractions. These results suggest that Tt-PCu_A_C and AniA do not interact, and that Cu transfer *via* oxidation or dissociation of the Cu(i) ion from Tt-PCu_A_C (and, thus, AccA) into the solvent is negligible, as expected from its sub-femtomolar dissociation constant (Fig. 7A). To test the function of Tt-PCu_A_C in *N. gonorrhoeae*, we replaced the *accA* open-reading frame with a gene encoding the soluble domain of Tt-PCu_A_C fused to the AccA N-terminal leader peptide. Transcription of the *pcu*_*A*_*C* gene was verified by qRT-PCR (Fig. S12). The resulting Δ*accA*/*pcu*_*A*_*C*^*+*^ mutant strain phenocopied the Δ*accA* mutant strain, as it did not grow or consume nitrite unless Cu was added to the cultures (Fig. 7C and D). These results indicate that Tt-PCu_A_C cannot functionally replace AccA in *N. gonorrhoeae*.

### AniA binds Cu(i) more weakly than AccA

**Fig. 8:**
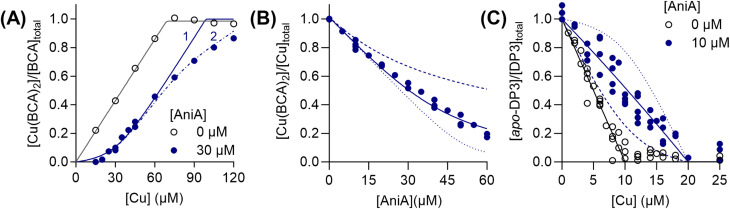
Cu(i)- and Cu(ii)-binding affinities of AniA. (A) Competition curves between: (A) BCA (138 µM) and AniA (0 or 30 µM) for Cu(i), (B) BCA (150 µM) and AniA for Cu(i) (53.5 µM), and (C) DP3 (10 µM) and AniA (0 or 10 µM) for Cu(ii). The competition approach is shown in Fig. 1A and 2A. Individual data points are shown. Competition curve fits (blue solid lines) produced the log *K*_D_ values shown in the text. Control curve fits (black solid lines), simulated fits for 10× lower (dotted lines) or 10× higher (dashed lines) *K*_D_ values, and simulated fits for different values for *n* (1 or 2; dot-dashed lines) are also shown.

For completeness, we determined the Cu(i)- and Cu(ii)-binding affinities of AniA. This protein competed with BCA (Fig. 8A), but not BCS, for Cu(i). Although AniA has two Cu-binding sites, namely the T1Cu and T2Cu sites, only one competed effectively with BCA (Fig. 8A and B), leading us to estimate a log *K*_D_ = −14.7 ± 0.1 M for this site. A meaningful Cu(i)-binding affinity for the second site could not be reliably estimated from the competition curve with BCA or a separate curve with Fz, suggesting that it is weak. Similarly, only one site in AniA competed with DP3 for Cu(ii) (Fig. 8C), yielding a log *K*_D_ = −12.4 ± 0.1 M. This site is likely the T1Cu site because titration of up to 1 eq. of Cu_aq_^2+^ into *apo*-AniA produced the T1Cu signal (Fig. S11). We assumed that the site that competed with BCA for Cu(i) is also the T1Cu site. Regardless of its precise identity, the competitive site would be the site that is relevant for receiving Cu(i) or Cu(ii) from a cellular Cu source such as AccA. Measured under the same conditions, the Cu(i)-binding affinity for this site is ≥50 times as weak as that for AccA, while the Cu(ii)-binding affinity is essentially indistinguishable to that of AccA (*cf.*Fig. 1 and 2).

### Cu(i) transfers from AccA to AniA against a favourable thermodynamic gradient

**Fig. 9:**
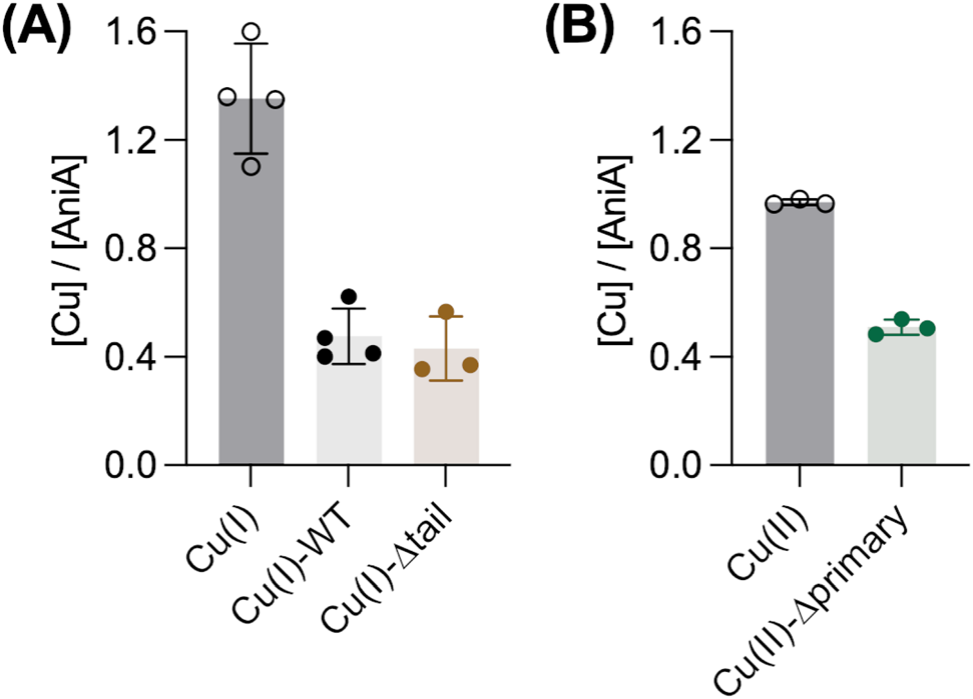
Transfer of Cu(i) or Cu(ii) from AccA to AniA. Amounts of Cu detected in AniA after incubation with 2 eq. of: (A) “free” Cu(i) and the Cu(i)-loaded forms of WT-AniA or Δtail-AccA under anaerobic conditions, or (B) “free” Cu(ii) and the Cu(ii)-loaded form of Δprimary-AccA under aerobic conditions. Data points from individual replicates (*N* = 3) are shown. Columns and error bars represent means and ±SD, respectively.

Because AccA has a higher Cu(i)-binding affinity than AniA, it was unclear whether Cu(i) transfer from AccA to AniA would occur. In our earlier experiments (Fig. 6), AccA was loaded with both Cu(i) and Cu(ii). Therefore, we did not know which Cu ion was transferred. To assess transfer of Cu(i), we loaded AccA with only Cu(i). We used sub-stoichiometric amounts (≤0.9 eq.) to ensure that Cu(i) was bound only to the high-affinity primary site and not to the low-affinity site in the tail. All loading, transfer, and chromatography steps were done anaerobically to prevent oxidation of Cu(i). Fig. 9A shows that ∼0.4 eq. of Cu was detected in AniA, indicating that WT-AccA can transfer Cu(i) to AniA. A similar result was obtained using Cu(i)-loaded Δtail-AccA, which retained an intact primary site (Fig. 9A). These results confirmed that Cu(i) is transferred from the primary site of AccA to AniA against a favourable thermodynamic gradient.

Finally, in our earlier experiments (Fig. 6), ∼1 eq. of total Cu was detected in AniA. Thus, in addition to Cu(i) from the primary site, AccA may also transfer Cu(ii) from the tail to AniA. To test this proposal, Δprimary-AccA was loaded with ≤0.9 eq. of Cu(ii), ensuring that Cu(ii) was bound to the high-affinity site in the tail and not elsewhere in the protein. After incubation with *apo*-AniA and separation of the two proteins, ∼0.5 eq. of Cu detected in AniA-containing fractions (Fig. 9B). Altogether, these results supported our earlier proposal that Cu from any site in purified AccA can be transferred to purified AniA.

## Conclusions

Extracytoplasmic Cu metallochaperones are key components of bacterial Cu delivery and homeostasis.^15,18^ Some supply Cu to Cu exporters that detoxify Cu when cellular Cu availability is high.^37–39^ Thus, metallochaperone-deficient mutant cells grow poorly in Cu-supplemented cultures.^38,40–42^ Others supply Cu to Cu importers or client cuproenzymes when cellular Cu availability is low.^15,18,43^ As shown here for *N. gonorrhoeae* and AccA, mutant cells lacking such metallochaperones show poor growth and/or target cuproenzyme activity without supplemental Cu.^19,22–24^

This phenotypic rescue by supplemental Cu is puzzling.^15^ The accepted thermodynamic model for Cu trafficking holds that Cu transfers between sites *via* fast, associative metal–ligand exchange reactions following a favourable, negative thermodynamic gradient, *i.e.* from higher to lower standard free energy of metalation 
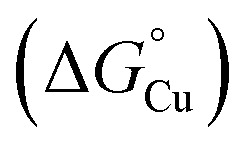
.^12^ If favourable gradients exist between: (1) the periplasmic Cu pool and metallochaperone, and (2) the metallochaperone and client cuproenzyme, then one also exists between the source and client cuproenzyme. Why, then, is a metallochaperone needed?

For metallochaperones that contribute to Cu detoxification, Cu binding by the metallochaperone likely lowers cellular Cu availability and, thus, suppresses potential mis-metalation and toxicity before Cu is exported.^44^ This explanation does not apply to metallochaperones that contribute to Cu acquisition. In conditions where Cu supply to cuproenzymes is insufficient, Cu availability is unlikely to be high enough to become toxic.^11^ We previously hypothesised that a high kinetic barrier prevents direct transfer of Cu from the source to some cuproenzymes.^15^ Our work here led us to formulate an alternative model that applies even if this barrier is low.

**Fig. 10:**
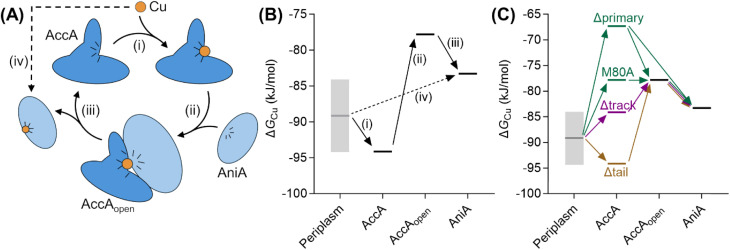
Thermodynamic model for AccA function. (A) Schematic and (B and C) free energy diagrams for transfer of Cu(i): (i) from the periplasmic milieu to AccA, (ii) dissociation of at least one primary site ligand in AccA (AccA_open_) upon contact with AniA, (iii) transfer of Cu(i) to AniA, and (iv) direct transfer of Cu(i) from the periplasmic milieu to AniA. The range of likely 
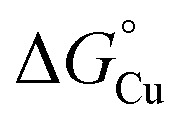
 values for the periplasmic milieu, shown as grey box, was estimated to be between the 
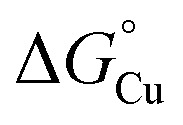
 values for WT-AccA and Δtrack-AccA. For clarity of presentation, the mid-point of this range is indicated. 
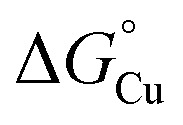
 values for AccA, AccA_open_, and AniA were calculated from the Cu(i)-binding affinities for WT-AccA, M80A-AccA, and the soluble domain of AniA, respectively (Table 1).

The culture phenotypes of *N. gonorrhoeae* mutant strains firmly established that only the Cu(i)-binding primary site of AccA, and not the Cu(ii)-binding C-terminal tail, is essential for activating AniA in cells (Fig. 5). Therefore, the key thermodynamic gradient to be considered would be between the Cu(i)-binding sites in both proteins. As in other PCu_A_C proteins, the primary site binds Cu(i) with a full first coordination sphere (Fig. 3B and S2). For Cu(i) to transfer, at least one ligand from AniA must displace at least one primary site ligand from AccA. Similar mechanisms were proposed for detoxification metallochaperones, *e.g.*, CusF from *E. coli* and CopC from *Pseudomonas syringae*,^45–49^ although the precise reaction pathways are undefined. Interactions with AniA may drive at least one ligand in the AccA primary site to dissociate (Fig. 10A). The relevant thermodynamic gradient for Cu(i) transfer would thus be setup between a three (or less)-coordinate primary site and AniA (Fig. 10A(iii) and B(iii)). Our estimates of the Cu(i)-binding affinities of H69A-AccA, M80A-AccA, H103A-AccA, M105A-AccA, and AniA (Fig. 1G and 8) confirm that this gradient is favourable.

The Δtrack-AccA and Δtail-AccA proteins, which retained an intact primary site (Fig. 1G), would follow the reaction pathway for WT-AccA (Fig. 10C). Meanwhile, Δprimary-AccA bound Cu(i) more weakly (*i.e.* with higher 
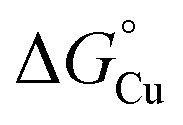
) than variants lacking only one (or two) primary site residue (Fig. 1G and S2B), making the gradient for Cu(i) transfer to AniA more favourable (Fig. 10C). However, these lower affinity Cu(i)-binding AccA variants were less effective at activating AniA in *N. gonorrhoeae* cells (Fig. 5 and S9). This finding can be explained if we consider the source of periplasmic Cu(i). It is not known whether an effective metal buffer operates in the periplasm. Nevertheless, Cu(i) is likely not “free”, but rather bound to the periplasmic milieu. Binding of Cu(i) to AccA would be driven by the thermodynamic gradient between this milieu and the four-coordinate primary site (Fig. 10A(i) and B(i)). This gradient would be less favourable for the weaker-binding variants (Fig. 10C).

We do not currently have a direct measure of 
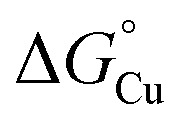
 for the periplasmic Cu(i) milieu. This would require examination of periplasmic Cu(i)-sensing transcriptional regulators,^11^ such as CusRS,^50^ CopRS,^51^ or CutF,^52^ but none has been identified in *Neisseria*. Nevertheless, the *N. gonorrhoeae* Δtrack-*accA* mutant strain may provide a clue. This mutant strain grew, albeit slowly, in the presence of nitrite (Fig. 5) and it was highly sensitive to inhibition by BCS (Fig. S10B and D). Based on these observations, 
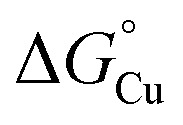
 for the periplasmic milieu is likely just below that for Δtrack-AccA (Fig. 10B and C). In other words, the apparent availability of Cu(i) in the *N. gonorrhoeae* periplasm, at least under our experimental conditions, is likely <10^−15^ M. This value is <10-fold lower than the ∼10^−14^ M estimate for *Pseudomonas aeruginosa*, based on characterisation of CopRS.^51^ Considering that CopRS controls the response to high cellular Cu, this ∼10^−14^ M estimate likely represents Cu-replete conditions, in which Cu supply to cuproenzymes would be sufficient.

Finally, comparing 
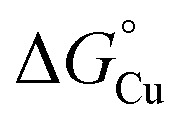
 values for the binding of Cu(i) to AccA, AniA, and the periplasmic milieu helps us understand why AccA is essential in cells when Cu supply is low but dispensable when Cu is high.^21–24,26^ The 
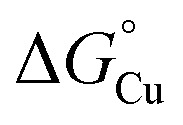
 value for AniA exceeds that predicted for the milieu, making direct insertion of Cu(i) from the milieu thermodynamically unfavourable (Fig. 10B(iv)). Adding Cu likely raises the availability of Cu(i) in the periplasm and, thus Cu(i) may transfer to AniA because of mass action. However, these conditions may also promote Cu(i) transfer between the milieu and non-cognate binding sites, causing mismetalation. Indeed, while adding 10 µM of Cu rescued nitrite consumption in *N. gonorrhoeae* mutant strains, it also mildly suppressed growth of the *accA*^*+*^ strain compared with the untreated control (Fig. 4B).

To answer our earlier question, we propose that the AccA metallochaperone is needed to scavenge and subsequently transfer Cu from the available Cu pool in the periplasm to the client cuproenzyme AniA, which cannot access the pool directly due to an unfavourable thermodynamic gradient (Fig. 10). This function for AccA expands the role of PCu_A_C-family metallochaperones beyond Cu_A_ assembly. Precisely how AccA overcomes the energetic penalty for this unfavourable transfer remains to be seen. Nevertheless, not all AniA or NirK-containing bacteria also have an AccA or PCu_A_C.^15^ Instead of relying on an alternative, unidentified Cu metallochaperone, 
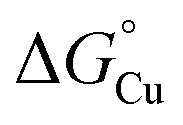
 for the periplasmic milieu in these bacteria may simply be high enough or, alternatively, 
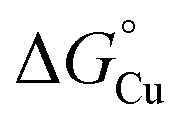
 for AniA may be low enough to create a favourable gradient between the two. Future work using the AccA–AniA system promises to reveal broader principles of periplasmic metallochaperone function.

## Experimental

### Cu-binding studies

All steps used MOPS buffer (50 mM, pH 7.2). Concentrations of *apo*-proteins were estimated using their theoretical *ε*_280_ values (AccA, 6990 cm^−1^ M^−1^; Tt-PCu_A_C, 8480 cm^−1^ M^−1^; and AniA, 24 870 cm^−1^ M^−1^; ExPASy ProtParam). Concentrations of *apo*-BCS, BCA, and Fz were estimated by titration with Cu_aq_^2+^ solutions of known concentrations and sodium ascorbate (2 mM), measuring absorbances at the relevant *λ*_max_ (Table S6). Data were fitted to the relevant equilibrium models using DynaFit (Biokin Ltd; Dataset S1). The sharp endpoints corresponded to the concentrations of the reporters, after considering the relevant Cu:reporter binding ratios. Concentrations of DP3 and DP2 were estimated using the same approach but without ascorbate and by measuring fluorescence emission at 550 nm following excitation at 350 nm.

Cu(i) binding affinities were estimated by competition with BCS, BCA, or Fz (Table S6).^32^ A master mix containing the competitor, *apo*-protein, and sodium ascorbate (2 mM) was prepared and added to a series of concentrated Cu_aq_^2+^ stocks (10×) in UVette™ cuvettes (Eppendorf). Absorbance spectra (400–800 nm) were recorded and absorbance intensities at the relevant *λ*_max_ (Table S6) were plotted against Cu concentrations. Curve fitting (Dataset S1) was performed in DynaFit to obtain *K*_D_ values. Cu(ii) binding affinities were estimated by competition with fluorometric reporters DP2 and DP3^33^ as above but without ascorbate and in black µClear® microplates (Greiner). Fluorescence emission spectra (450–700 nm) following excitation at 350 nm were recorded and emission intensities at 550 nm were used to obtain *K*_D_ values (Dataset S1).

### X-ray crystallography

The AccA protein used for crystallisation was from *N. meningitidis* serogroup B (UniProt: Q9JYJ5), which differs from AccA from *N. gonorrhoeae* (UniProt A0AAQ1E0N0) at positions 38 (Ile instead of Met) and 139 (Ile instead of Thr). Cu-free and Cu-loaded AccA proteins (10 mg mL^−1^ each) were used to set up commercial sparse matrix crystallisation screens using a Mosquito liquid handler (SPT Labtech) at The University of Queensland Remote Operation Crystallisation and X-ray (UQ-ROCX) Diffraction Facility, Centre for Microscopy and Microanalysis. After incubation at 20 °C for 30 days, conditions producing crystals were identified (0.2 M NaCl, 25 w/v% PEG3350, 0.1 M Tris, pH 8.5) and further optimised by varying polyethylene glycol (PEG) concentrations and pH of the buffers to produce diffraction quality crystals. Crystals were cryoprotected using 20 v/v% glycerol in the crystallisation buffer and flash-cooled in liquid nitrogen.

Diffraction data were collected on the MX1 beamline, Australian Synchrotron, using 0.5° oscillations with a total rotation angle of 180°.^53^ Diffraction data were collected at the Cu Kα edge wavelength at approximately 8979 eV, with the intent to maximise the anomalous signal from the Cu ions. The data were indexed and integrated using XDS,^54^ then scaled and merged using the AIMLESS software from the CCP4 suite.^55^ During this process, images with radiation damage were identified and excluded during the scaling and merging. Initial phases were obtained using single wavelength anomalous diffraction (SAD) and the software Autosol.^56^ Initial model building was carried out using Phenix.AutoBuild,^57^ followed by iterative model building using Coot^58^ and refinement using Phenix.Refine.^57^ Statistics for crystallographic data collection and refinement are shown in Table S7.

### *N. gonorrhoeae* culture conditions

*N. gonorrhoeae* (Table S1) was cultured on GC agar (Oxoid) or in GC broth, both with Kellogg's supplements I and II.^59^ GC broth also contained sodium bicarbonate (0.042 w/v%). Incubation was always at 37 °C. Antibiotics were used only for making mutant strains. GC agar plates were incubated (16–24 h) with atmospheric CO_2_ (5–9 v/v%; Oxoid CO_2_ Gas Generator Sachet) in an air-tight container (2.5 L). Bacteria from overnight agar plates were resuspended in PBS and used to inoculate GC broth to OD_600_ = 0.1. Broth cultures (30 mL in 50 mL screw-capped tubes) were not shaken and sodium nitrite (2 mM) was added at 0, 5, 7, and 8 h. The Δ*aniA* mutant strain^26^ did not grow even with supplemental Cu, confirming that these conditions were O_2_-limiting.

### Nitrite consumption assays

After 8 h of growth in GC broth, bacteria were centrifuged (4000×*g*, 4 °C, 5 min), resuspended in GC broth (1 mL) with glycerol (20 v/v%), and stored (−80 °C) until further use. Frozen cells were thawed on ice, centrifuged (7000×*g*, 1 min), resuspended in GC broth (0.5 mL), and split into two aliquots (0.25 mL each). One aliquot was used for measuring total protein content (SI), while the other was placed immediately in a dry bath (37 °C, 10 min). After adding sodium nitrite (1.5 mM), the reaction mixture was sampled (5 µL each) at 0 min and every minute up to 10 min, diluted in deionised water (95 µL), and mixed with Griess reagent (100 µL; Merck). The concentration of nitrite at each time-point was determined by comparing solution absorbances at 545 nm against a standard curve of sodium nitrite (0–160 µM). Nitrite concentrations decreased linearly with time and rates of nitrite consumption were obtained by normalising the reaction slopes to total cellular protein content.

### Cu transfer assays

To prepare Cu(i)- and Cu(ii) loaded forms of AccA and the Cu(i)-loaded form of Tt-PCu_A_C, the *apo*-metallochaperones were incubated (15 min) with excess Cu_aq_^2+^ (>3 eq.) and sodium ascorbate (2 mM), and desalted on a PD-10 column (Cytiva). The Cu(ii)-loaded form of Δprimary-AccA was prepared as above, but with only 0.9 eq. Cu_aq_^2+^. Likewise, the Cu(i)-loaded forms of WT-AccA and Δtail-AccA were prepared with 0.9 eq. Cu_aq_^2+^, but all steps were performed in the anaerobic chamber. The concentration of Cu in each metallochaperone sample was determined by ICP MS or by using excess BCS with and without ascorbate to determine the redox status of Cu.

All Cu transfer assays were performed in Tris–HCl (50 mM, pH 7.4, 150 mM NaCl, 15 v/v% glycerol). Cu or the relevant *holo*-metallochaperone (2–3 eq.) was added to *apo*-AniA (50–70 µM). In experiments where Cu transfer was monitored using the AniA T1Cu signal, solution absorbances were measured for up to ∼2 h at room temperatures. Otherwise, amounts of Cu bound to AniA were measured by ICP MS following separation of the two proteins on an anion exchange column. AccA and Tt-PCu_A_C were found in the flowthrough. AniA was bound to the column and eluted with 350 mM NaCl. In experiments that assessed transfer of Cu(i), all steps were performed in the anaerobic chamber.

The concentration of *holo*-AniA and/or *holo*-metallochaperones in every sample before and after transfer was determined by measuring solution absorbances at 280 nm. Samples containing *holo*-metallochaperones were first made *apo* by incubating in guanidine hydrochloride (6 M) and EDTA (1 mM).

## Author contributions

Author initials in this section are listed in alphabetical order. SF generated and characterised all *N. gonorrhoeae accA* mutant strains. SF, WE generated and characterised the *N. gonorrhoeae accA*/*pcu*_*A*_*C*^*+*^ mutant strain. CO, DT, KD, SF, WE, YH generated and characterised AccA proteins. SF, WE generated and characterised Tt-PCu_A_C and AniA proteins. SF, WE examined Cu transfer between AccA and AniA. WE examined Cu transfer between Tt-PCu_A_C and AniA, and measured AniA affinities. BK, CJ, DN, DT, ZL determined the X-ray crystal structure of AccA. CO, GL, SF characterised the synthetic tail peptide. KD measured gene transcription levels. KD, SF, WE drafted the manuscript. All authors contributed to editing and proofing the manuscript. AM, BK, KD conceived the project. KD, with help from SF, coordinated and had overall responsibility for the research program.

## Conflicts of interest

The authors declare no competing interests.

## Supplementary Material

SC-OLF-D5SC08738D-s001

SC-OLF-D5SC08738D-s002

## Data Availability

Data supporting this work are available either in the main article or supplementary information (SI). DynaFit scripts used for estimating binding constants in Fig. 1, 2, 7A, and 8 have been uploaded as part of the SI (Dataset S1). Crystallographic data for Cu-loaded AccA protein in Fig. 3 have been deposited to the PDB under the PDB ID: 9YAH and can be obtained from https://www.rcsb.org/structure/9YAH. Supplementary information: SI text, Fig. S1–S13, Tables S1–S7 and Dataset S1. See DOI: https://doi.org/10.1039/d5sc08738d.
