# Supplementary material for: AccA from Neisseria gonorrhoeae provides a new framework for understanding periplasmic copper metallochaperones
Source: Chem Sci. 2026 Mar 12;17(18):9270–84. doi: 10.1039/d5sc08738d (PMC13006917; doi:10.1039/d5sc08738d)
Supplement: SC-017-D5SC08738D-s001 [file SC-017-D5SC08738D-s001.pdf]

Supplementary Information (SI) for Chemical Science.
This journal is © The Royal Society of Chemistry 2026

Supplementary Dataset S1. DynaFit scripts used in this work.

1. Competition between BCS and AccA for Cu(I). Used to generate Figure 1B.
2. Competition between Fz and ∆primary-AccA for Cu(I). Used to generate Figure 1C.
3. Competition between BCS and ∆track-AccA for Cu(I). Used to generate Figure 1D.
4. Competition between BCS and ∆tail-AccA. Used to generate Figure 1E.
5. Competition between Fz and the synthetic tail peptide. Used to generate Figure 1F.
6. Competition between DP-3 and WT-AccA for Cu(II). Used to generate Figure 2B.
7. Competition between DP-3 and ∆primary-AccA for Cu(II). Used to generate Figure 2C.
8. Competition between DP-3 and ∆track-AccA for Cu(II). Used to generate Figure 2D.
9. Competition between DP-3 and ∆tail-AccA for Cu(II). Used to generate Figure 2E.
10. Competition between DP-3 and ∆tail-AccA for Cu(II). Used to generate Figure 2E.
11. Competition between BCS and Tt-PCuAC. Used to generate Figure 8A.
12. Competition between BCA and AniA. Used to generate Figure 9A.
13. Competition between BCA and AniA. Used to generate Figure 9B.
14. Competition between DP3 and AniA. Used to generate Figure 9C.

1. **Competition between BCS and AccA for Cu(I). Used to generate Figure 1B.**

```
[Model]
2 BCS monomers bind 1 Cu(I).
WT-AccA binds 1 Cu(I) per monomer.

[Components]
; P = WT-AccA (protein)
; L = BCS (Ligand)
; M = Cu(I) (metal)

[task]
task = fit
data = equilibria

[mechanism]
M + L + L <==> MLL       :  Kd1 dissociation
M + P <==> MP            :  Kd2 dissociation

[concentrations] micromolar
L = 75
P = 20

[constants] micromolar
Kd1 = 1.58489E-08
Kd2 = 1e-011?

[responses] MLL
MLL  = 0.0267
[data]
variable    M
set         Titration

[output]
directory ./WT-AccA_Cu(I)/

[set:Titration]

M     MLL
0     -0.00494
5     0.02716
10    0.074074
15    0.148148
20    0.204938
25    0.261728
30    0.4
35    0.491358
40    0.62963
50    0.911111
60    1
70    0.940741
0     0.002809
5     0.039326
10    0.075843
15    0.143258
20    0.185393
25    0.241573
30    0.438202
35    0.536517
40    0.744382
50    1
```

```
60    0.926966
70    0.859551

[end]
```

2. Competition between Fz and ∆primary-AccA for Cu(I). Used to generate Figure 1C.

```
[Model]
2 Fz monomers bind 1 Cu(I).
ΔPrimary-AccA binds 1 Cu(I) per monomer.

[Components]
; P = ΔPrimary-AccA (Protein)
; L = Fz (Ligand)
; M = Cu(I) (Metal)

[task]
task = fit
data = equilibria

[mechanism]
M + L + L <==> MLL       :  Kd1 dissociation
M + P <==> MP            :  Kd2 dissociation

[concentrations] micromolar
L = 142
P = 30

[constants] micromolar
Kd1 = 0.000769231
Kd2 = 1e-5?

[responses] MLL
MLL  = 0.0139

[data]
variable    M
set         Titration

[output]
directory ./primary-AccA_Cu(I)/

[set:Titration]
M     MLL
0     0
5     0.045317
10    0.081571
15    0.120846
20    0.151057
25    0.187311
30    0.220544
35    0.244713
40    0.287009
60    0.392749
80    0.516616
100   0.63142
120   0.734139
140   0.809668
160   0.870091
180   0.936556
200   0.963746
```

```
220   0.981873
240   1

[end]
```

3. Competition between BCS and ∆track-AccA for Cu(I). Used to generate Figure 1D.

```
[model]
2 BCS monomers bind 1 Cu(I).
Δtrack-AccA binds 1 Cu(I) per monomer.

[components]
; P = Δtrack-AccA (Protein)
; L = BCS (Ligand)
; M = Cu(I) (Metal)

[task]
task = fit
data = equilibria

[mechanism]
M + L + L <==> MLL      :  Kd1 dissociation
M + P <==> MP           :  Kd2 dissociation

[concentrations] micromolar
L = 81.1
P = 30

[constants] micromolar
Kd1 = 1.58489E-08
Kd2 = 1e-08?

[responses] MLL
MLL  = 0.0247

[data]
variable    M
set         Titration

[output]
directory ./track-AccA_Cu(I)/

[set:Titration]
M     MLL
5     0.123223
10    0.225118
15    0.308057
25    0.509479
30    0.609005
35    0.739336
40    0.798578
70    0.964455
80    1
0     0.002183
5     0.09607
10    0.218341
15    0.30131
25    0.434498
30    0.60262
35    0.60917
40    0.722707
```

```
50    0.842795
70    0.984716
80    1

[end]
```

4. Competition between BCS and ∆tail-AccA. Used to generate Figure 1E.

```
[model]
2 BCS monomers bind 1 Cu(I).
∆tail-AccA binds 1 Cu(I) per monomer.

[components]
; P = AccA
; L = BCS
; M = Cu

[task]
task = fit
data = equilibria

[mechanism]
M + L + L <==> MLL      :  Kd1 dissociation
M + P <==> MP           :  Kd2 dissociation

[concentrations] micromolar
L = 73
P = 30

[constants] micromolar
Kd1 = 1.58489E-08
Kd2 = 1e-011?

[responses] MLL
MLL  = 0.0273

[data]
variable    M
set         Titration

[output]
directory ./tail-AccA_Cu(I)/

[set:Titration]
M     MLL
0     0.002053
5     0.01232
10    0.043121
15    0.073922
20    0.119097
25    0.193018
30    0.244353
35    0.338809
40    0.445585
50    0.731006
60    0.870637
70    1
80    0.977413
0     0.002041
5     0.018367
10    0.030612
```

```
15    0.069388
20    0.112245
25    0.171429
30    0.244898
35    0.332653
40    0.453061
50    0.761224
60    0.879592
70    1
80    0.967347

[end]
```

5. Competition between Fz and the synthetic tail peptide. Used to generate Figure 1F.

```
[model]
2 Fz monomers bind 1 Cu(I).
1 tail peptide monomer binds 1 Cu(I).

[components]
; P = tail peptide (protein)
; L = Fz (ligand)
; M = Cu(I) (metal)

[task]
task = fit
data = equilibria

[mechanism]
M + L + L <==> MLL       :  Kd1 dissociation
M + P <==> MP            :  Kd2 dissociation

[concentrations] micromolar
L = 82.5
P = 30

[constants] micromolar
Kd1 = 0.000769231
Kd2 = 1e-6?

[responses] MLL
MLL  = 0.0242

[data]
variable    M
set         Titration

[output]
directory ./tail_peptide_Cu(I)/

[set:Titration]
M     MLL
100   0.998278
90    1
80    0.980552
70    0.741312
60    0.719569
50    0.62877
40    0.420136
30    0.41867
20    0.198686
```

```
10    0.104062
0     -0.01243
150   1.036894
110   1
95    0.846158
85    0.675084
75    0.65751
65    0.693511
55    0.691748
45    0.586077
35    0.510095
25    0.252858
15    0.267247
5     0.140875
0     0.015299
100   0.948037
90    1
80    0.813106
70    0.652264
60    0.682911
50    0.548626
40    0.480729
30    0.269432
20    0.109381
10    0.115051
0     0.013924

[end]
```

6. **Competition between DP-3 and WT-AccA for Cu(II). Used to generate Figure 2B.**

```
[model]
1 DP-3 monomer binds 1 Cu(II).
1 WT-AccA monomer binds 1 Cu(II).

[Components]
; P = WT-AccA (protein)
; L = DP-3 (Ligand)
; M = Cu(II) (metal)

[task]
task = fit
data = equilibria

[mechanism]
M + L <==> ML           :  Kd1 dissociation
M + P <==> MP           :  Kd2 dissociation

[concentrations] micromolar
L = 4
P = 4

[constants] micromolar
Kd1 = 3.98107E-07
Kd2 = 6.4e-005?

[responses] L
L  = 0.25

[data]
variable    M
```

```
set         Titration

[output]
directory ./WT-AccA_Cu(II)/

[set:Titration]
M     L
0     1
1     0.890530223
2     0.760017962
3     0.577120974
4     0.45623439
5     0.365792963
6     0.260801518
7     0.204835544
8     0.130512261
9     0.123572772
10    0.055069902
12    0.034922476
15    0
20    -0.040294852
25    -0.013655973
0     1
1     0.817894
2     0.66726
3     0.611441
4     0.463381
5     0.377276
6     0.280285
7     0.25099
8     0.204276
9     0.146477
10    0.121536
12    0.0865
15    0.0865
20    -0.00594
25    0

[end]
```

7. Competition between DP-3 and ∆primary-AccA for Cu(II). Used to generate Figure 2C.

```
[model]
1 DP-3 monomer binds 1 Cu(II).
1 ∆primary-AccA monomer binds 1 Cu(II).

[components]
; P = ∆primary-AccA (protein)
; L = DP-3 (ligand)
; M = Cu(II) (metal)

[task]
task = fit
data = equilibria

[mechanism]
M + L <==> ML            :  Kd1 dissociation
M + P <==> MP            :  Kd2 dissociation

[concentrations] micromolar
L = 4
```

```
P = 4

[constants] micromolar
Kd1 = 3.98107E-07
Kd2 = 1e-5?

[responses] L
L  = 0.25

[data]
variable    M
set         Titration

[output]
directory ./primary-AccA_Cu(II)/

[set:Titration]
M     L
0     1
1     0.874326
2     0.615185
3     0.457542
4     0.308292
5     0.203996
6     0.114486
8     0.032168
10    0.027373
12    0.002997
15    0
0     1
1     0.84328
2     0.687445
3     0.484969
4     0.36008
5     0.212202
6     0.143236
7     0.068081
8     0.033378
10    0.009947
12    -0.00066
15    0

[end]
```

8. Competition between DP-3 and ∆track-AccA for Cu(II). Used to generate Figure 2D.

```
[model]
1 DP-3 monomer binds 1 Cu(II).
1 ∆track-AccA monomer binds 1 Cu(II).

[components]
; P = ∆track-AccA (protein)
; L = DP-3 (ligand)
; M = Cu(II) (metal)

[task]
task = fit
data = equilibria

[mechanism]
M + L <==> ML            :  Kd1 dissociation
```

```
M + P <==> MP            :  Kd2 dissociation

[concentrations] micromolar
L = 4
P = 4

[constants] micromolar
Kd1 = 3.98107E-07
Kd2 = 1.57e-007?

[responses] L
L  = 0.25

[data]
variable    M
set         Titration

[output]
directory ./track-AccA_Cu(II)/

[set:Titration]
M     L
0     1
1     0.816175
2     0.667836
3     0.565092
4     0.401073
5     0.300805
6     0.135754
7     0.085207
8     0.049309
10    0.034454
12    0.013204
15    0
0     1
1     0.964182
2     0.811229
3     0.566554
4     0.445789
5     0.320426
6     0.155615
7     0.115198
8     0.065828
10    0.012585
12    0.00484
15    0

[end]
```

9. Competition between DP-3 and ∆tail-AccA for Cu(II). Used to generate Figure 2E.

```
[model]
1 DP-3 monomer binds 1 Cu(II).
1 ∆tail-AccA monomer binds 1 Cu(II).

[components]
; P = ∆tail-AccA (protein)
; L = DP-3 (Ligand)
; M = Cu(II) (metal)

[task]
```

```
task = fit
data = equilibria

[mechanism]
M + L <==> ML            :  Kd1 dissociation
M + P <==> MP            :  Kd2 dissociation

[concentrations] micromolar
L = 4
P = 4

[constants] micromolar
Kd1 = 3.98107E-07
Kd2 = 6.4e-007?

[responses] L
L  = 0.25

[data]
variable    M
set         Titration

[output]
directory ./tail-AccA_Cu(II)/

[set:Titration]
M     L
0     1
1     0.838348
2     0.587466
3     0.285951
4     0.101888
5     0.030504
6     0.037145
7     0.010583
8     0.022619
10    0.029674
12    0.015978
15    0
0     1
1     0.867506
2     0.57474
3     0.327338
4     0.121303
5     0.046363
6     0.027978
7     0.027378
8     0.019185
10    0.023381
12    0.018585
15    0

[end]
```

10. Competition between DP-3 and ∆tail-AccA for Cu(II). Used to generate Figure 2E.

```
[model]
1 DP-3 monomer binds 1 Cu(II).
1 synthetic tail peptide monomer binds 1 Cu(II).

[components]
```

```
; P = tail peptide (protein)
; L = DP-3 (ligand)
; M = Cu(II) (metal)

[task]
task = fit
data = equilibria

[mechanism]
M + L <==> ML           :  Kd1 dissociation
M + P <==> MP           :  Kd2 dissociation

[concentrations] micromolar
L = 4
P = 4

[constants] micromolar
Kd1 = 3.98107E-07
Kd2 = 1.57e-007?

[responses] L
L  = 0.25

[data]
variable    M
set         Titration

[output]
directory ./tail_peptide_Cu(II)/

[set:Titration]
M     L
0     1
1     0.830524
2     0.630918
3     0.40208
4     0.234935
5     0.141499
6     0.121234
7     0.060617
8     0.079089
10    0.046628
12    0.003587
15    0
0     1
1     0.792706
2     0.620162
3     0.433167
4     0.275245
5     0.1722
6     0.110958
7     0.056597
8     0.046104
10    0.028213
12    0.039394
15    0

[end]
```

11. Competition between BCS and Tt-PCuAC. Used to generate Figure 8A.

```
[model]
2 BCS monomers bind 1 Cu(I).
Tt-PCuAC binds 1 Cu(I) per monomer.

[components]
; P = Tt-PCuAC (protein)
; L = BCS (ligand)
; M = Cu(I) (metal)

[task]
task = fit
data = equilibria

[mechanism]
M + L + L <==> MLL       :  Kd1 dissociation
M + P <==> MP            :  Kd2 dissociation

[concentrations] micromolar
L = 98.5
P = 30

[constants] micromolar
Kd1 = 1.58489E-08
Kd2 = 6.5e-011?

[responses] MLL
MLL  = 0.0203

[data]
variable    M
set         Titration

[output]
directory ./PCuAC_Cu(I)/

[set:Titration]
M     MLL
113.25      0.725288961
98.15 0.784707728
90.6  0.963410246
83.05 1
75.5  0.889698156
67.95 0.815179998
60.4  0.718941171
52.85 0.623292502
45.3  0.538554546
37.75 0.456421919
30.2  0.349301167
22.65 0.191614008
15.1  0.165143293
7.55  0.05537403
113.25      0.773703526
98.15 0.817262578
90.6  0.874897412
83.05 1
75.5  0.792316388
67.95 0.804503012
60.4  0.736555227
52.85 0.617956302
```

```
45.3  0.461016739
37.75 0.383251521
30.2  0.285355921
22.65 0.202759411
15.1  0.126759473
7.55  0.058749748
113.25      0.785944542
98.15 0.881704988
90.6  0.925765793
83.05 1
75.5  0.954716981
67.95 0.657444046
60.4  0.741868459
52.85 0.585211214
45.3  0.512153388
37.75 0.384523719
30.2  0.308089527
22.65 0.208341609
15.1  0.119120006

[end]
```

12. Competition between BCA and AniA. Used to generate Figure 9A.

```
[model]
2 BCA monomers bind 1 Cu(I).
1 AniA monomer binds 1 Cu(I).

[components]
; L = BCA (ligand)
; M = Cu(I) (metal)
; P = AniA (protein)

[task]
task = fit
data = equilibria

[mechanism]
M + L + L <==> MLL       :  Kd1 dissociation
M + P <==> MP            :  Kd2 dissociation

[concentrations] micromolar
L = 138
P = 30

[constants] micromolar
Kd1 = 6.25E-06
Kd2 = 1e-10?

[responses] MLL
MLL  = 0.0145

[data]
variable    M
set         Titration

[output]
directory ./BCAvsAniAvaryCu/

[set:Titration]
M     MLL
```

```
0     -0.012696429
5     -0.011178571
10    -0.003785714
15    0.001160714
15    -0.000839286
20    0.016464286
25    0.073642857
30    0.099892857
30    0.081678571
35    0.172446429
40    0.222875
40    0.245482143
45    0.280910714
60    0.457410714
75    0.591553571
90    0.707589286
105   0.801410714
120   0.865535714

[end]
```

13. Competition between BCA and AniA. Used to generate Figure 9B.

```
[model]
2 BCA monomers bind 1 Cu(I).
1 AniA monomer binds 1 Cu(I).

[components]
; L = BCA (ligand)
; M = Cu(I) (metal)
; P = AniA (protein)

[task]
task = fit
data = equilibria

[mechanism]
M + L + L <==> MLL            :  Kd1 dissociation
M + P <==> MP           :  Kd2 dissociation

[concentrations] micromolar
L = 150
M = 53

[constants] micromolar
Kd1 = 6.25E-06
Kd2 = 1e-9?

[responses] MLL
MLL  = 0.0187?

[data]
variable    P
set         Titration

[output]
directory ./BCAvsAniAvaryAniA/

[set:Titration]
```

```
M      MLL
0      1.002062
10     0.821636
20     0.670956
30     0.515315
40     0.379225
50     0.256253
60     0.172995
0      0.996851
10     0.785206
50     0.264522
0      1.000929
10     0.80802
40     0.39411
0      1
5      0.91375406
10     0.85396771
15     0.74515113
20     0.68216501
25     0.60765974
30     0.55301762
35     0.49143448
40     0.43519248
45     0.36056414
50     0.32989564
55     0.25959929
60     0.19621936

[end]
```

14. Competition between DP3 and AniA. Used to generate Figure 9C.

```
[model]
1 DP-3 monomer binds 1 Cu(II).
1 AniA monomer binds 1 Cu(II).

[components]
; M = Cu(II) (metal)
; L = DP3 (ligand)
; P = AniA (protein)

[task]
task = fit
data = equilibria

[mechanism]
M + L <==> ML            :  Kd1 dissociation
M + P <==> MP            :  Kd2 dissociation

[concentrations] micromolar
L = 10
P = 10

[constants] micromolar
Kd1 = 3.98107E-07
Kd2 = 2.41e-006?

[responses] L
L  = 0.1

[data]
```

```
variable    M
set         Titration

[output]
directory ./DP3vsAniA/

[set:Titration]
M     L
0     1.044053012
2     0.963028916
4     0.752787952
6     0.608510843
8     0.545257831
10    0.405498795
12    0.310016867
14    0.241944578
16    0.144956627
0     1.096462651
2     0.94706506
4     0.827486747
6     0.753992771
8     0.6558
10    0.421763855
12    0.351583133
14    0.2808
16    0.240137349
0     1.420084337
3     1.111349398
6     0.875807229
9     0.695084337
12    0.461048193
15    0.287554217
18    0.180927711
25    0.126710843
0     1.196379518
2     1.118668675
4     1.037945783
6     0.864451807
8     0.727704819
10    0.472283133
12    0.481018072
14    0.362945783
16    0.304210843
18    0.198789157
25    0.087343373
30    0.103307229
0     0.997692771
4     0.709740964
8     0.491668675
10    0.441668675
12    0.271487952
16    0.126006024
20    0.030222892
25    0.014861446

[end]
```
