# Supplementary material for: AccA from Neisseria gonorrhoeae provides a new framework for understanding periplasmic copper metallochaperones
Source: Chem Sci. 2026 Mar 12;17(18):9270–84. doi: 10.1039/d5sc08738d (PMC13006917; doi:10.1039/d5sc08738d)
Supplement: SC-017-D5SC08738D-s002 [file SC-017-D5SC08738D-s002.pdf]

Supplementary Information (SI) for Chemical Science.
This journal is © The Royal Society of Chemistry 2026

Supplementary Information

AccA from *Neisseria gonorrhoeae* provides a framework for understanding periplasmic copper metallochaperones.

Samantha Firth, William Earl, Denis Thaqi, YoungJin Hong, Charlotte O'Hern, Gemma Luscombe, Dalton Heng Yong Ngu, Zhenyao Luo, Chacko Jobichen, Bostjan Kobe, Alastair McEwan, Karrera Djoko

Corresponding authors: Samantha Firth (samantha.j.firth@durham.ac.uk) and Karrera Djoko (karrera.djoko@durham.ac.uk)

This PDF file includes:

Supplementary Experimental
Figures S1 to S13
Tables S1 to S7
Supplementary References

Other supplementary materials for this manuscript include the following:

Dataset S1

Supplementary Experimental

General methods, reagents, and equipment. Chemicals were of analytical grade and supplied by Merck or Melford unless stated otherwise. Kits and reagents for molecular biology were from New England Biolabs. The synthetic tail peptide, DP2, and DP3 were synthesised commercially as acetate salts by GenScript. Deionised water was used in all procedures. Concentrations of Cu^{2+}_{aq} in solution were estimated using excess bathocuproine disulfonate (BCS; 1 mM) as a colourimetric reporter for Cu(I) and sodium ascorbate (2 mM) as a reducing agent(1). Spectrophotometry was performed using a Genesys 150 UV-Vis spectrophotometer (Fisher Scientific), Cary 3500 Compact UV-vis spectrophotometer (Agilent), SPECTROstar Nano plate reader (BMG Labtech), or Synergy H4 hybrid plate reader (Agilent BioTek).

***E. coli* culture conditions.** *E. coli* (Table S2) was cultured on LB agar or in LB medium and always supplemented with the appropriate antibiotics. Broth cultures were always shaken (180-200 rpm). Unless otherwise noted, all incubation steps were at 37 °C.

Protein overexpression constructs*.* Genes encoding AccA and Tt-PCu_AC proteins with and without the AccA leader peptide were amplified by PCR from the relevant Golden Gate-assembled plasmids (Table S3). All constructs were sub-cloned between the *Nde*I and *Bam*HI sites of pET-11b, except for genes encoding the Δtail-AccA variant and Tt-PCu_AC, which were sub-cloned into the *Stu*I site of pSATL. The gene encoding the soluble domain of *aniA* was amplified by PCR from *N. gonorrhoeae* 1291 genomic DNA and sub-cloned between the *Nco*I and *Xho*I sites of pET-29a. All constructs were propagated in *E. coli* DH5α and transformed into *E. coli* BL21 strains.

Protein overexpression and purification. All buffers are listed in Table S5. *E. coli* colonies from overnight agar plates were used to inoculate LB media (1 L in a 2 L flask) to OD_{600} = 0.01. Cultures were shaken until OD_{600} ~0.4-0.6 (~3 h), induced with isopropyl β-D-1-thiogalactopyranoside (IPTG; 0.1-0.4 mM), and supplemented with more antibiotics to maintain selection pressure. Cultures were cooled (room temperature) and shaken for 16 h, except for those expressing AniA, which were shaken for only 4 h. Bacteria were harvested (6000 x*g*, 4 °C, 15 min) and resuspended in Resuspension Buffer A (Table S5), except for those expressing Δtail-AccA variant and Tt-PCu_AC, which were resuspended in Resuspension Buffer B (Table S5). All bacteria were lysed by sonication using a Q700CA sonicator (45% amplitude, 10 min total; Qsonica), re-centrifuged (66000 x*g*, 4 °C, 15 min), and filtered through a 0.45 µm polyethersulfone syringe-driven unit (Starlab).

Proteins were purified from crude extracts using pre-packed chromatography columns (Cytiva) as outlined in Figure S14. AniA was expressed with a C-terminal 6xHis, which was removed by mixing (50 rpm, room temperature, overnight) with thrombin (10 U/mg) and glycerol (20 v/v %). Unlike previous preparations of AniA(4–6), Cu was omitted from all expression and purification steps. The Δtail-AccA and Tt-PCu_AC proteins were expressed with an N-terminal 6xHis-SUMO tag. This tag was removed by mixing (50 rpm, 4 °C, overnight) with hSENP2 protease (~1 mg) and glycerol (15 v/v %). All purified proteins were stored at -80 °C. Their identities (Table S6) were established using electrospray ionisation mass spectrometry in the positive ion mode (Mass Spectrometry Facility, Durham Chemistry, UK). All proteins were confirmed to be metal-free by inductively coupled plasma-mass spectrometry (ICP-MS; Bioanalytic Facility, Durham Biosciences, UK).

***N. gonorrhoeae* mutant constructs.** Mutant strains, genetic constructs, and primers are listed in Tables S2-S4. The base *accA* construct contained the 5' upstream flanking region of *accA* (1000 bp), *accA* (NGO_1215, 474 bp), a promoterless $spec^R$ cassette from pCTS32(2), and the 3' downstream flanking region of *accA* (1000 bp). The four DNA pieces were amplified by PCR (Q5 DNA Polymerase), subcloned into the *Sma*I site of pTRB479, propagated in *E. coli* DH5α, and assembled using *Bsa*I-HF NEBridge(R) Golden Gate. The assembled construct was subcloned into the *Hinc*II site of pTRB479, propagated in *E. coli* DH5α, and used to create the Δ*accA* (Q5 Site-Directed

Mutagenesis Kit) and the H69A-, M105A-, H103A/M105A-, and Δtail-*accA* (splice overlap extension PCR) mutant constructs. Genes encoding the M80A-, H103A-, H69A/M80A-, Δprimary-, and Δtrack-AccA, along with a codon-optimised gene encoding the soluble domain of Tt-PCu$_A$C (UniProt: Q5SGY7) fused to the AccA signal peptide, were synthesised commercially (Integrated DNA Technologies) and assembled as described above for wild-type *accA*.

Each assembled construct was subcloned into the *Sma*I site of pUC19, linearised by PCR, and transformed into *N. gonorrhoeae* 1291 following established procedures(3). Spectinomycin (100 µg/mL) was used to select at least three transformants. Homologous recombination was verified by PCR and Sanger sequencing (Genomics and Bioinformatics Facility, Durham Biosciences, UK). The three transformants were confirmed to have identical culture phenotypes.

Cellular protein content. The second aliquot of *N. gonorrhoeae* cells from nitrite consumption assays was centrifuged (21000 x*g*, 1 min) and resuspended in PBS (0.25 mL). Protein concentrations were determined using QuantiPro™ BCA Assay Kit (Merck).

RNA extraction and qRT-PCR. Bacterial pellets from broth cultures were resuspended immediately in RNAPro Solution (500 µL; MP Biomedicals) and stored (-80 °C) until further use. Bacteria were lysed in Lysing Matrix B and a FastPrep 24 G instrument (10 m/s, 30 s, 2 cycles) and total RNA was extracted following the manufacturer's protocol (MP Biomedicals). RNA extracts were treated with RNase-Free DNase I enzyme. Complete removal of gDNA was confirmed by PCR. The gDNA-free RNA was purified using Monarch RNA Cleanup Kit and visualized on an agarose gel.

cDNA was generated from RNA (1 µg) using the SuperScript IV First-Strand Synthesis System (Invitrogen). Quantitative reverse transcriptase PCR (qRT-PCR) was performed in 20 µL reactions using Luna Universal RT-qPCR Master Mix, cDNA (5 ng), and the appropriate primer pairs (0.4 µM each). Each sample was analysed in technical duplicates. Amplicons were detected in a CFX Connect Real-Time PCR Instrument (Bio-Rad Laboratories). Cq values were calculated using LinRegPCR(7) after correcting for amplicon efficiency. Cq values of technical duplicates were typically within ±0.25 of each other. The *gyrA* gene, which encodes DNA gyrase, was used as the reference gene.

Supplementary Figures

```
        10         20         30         40
MKKLLAAVMM AGLAGAVSAA GVHVEDGWAR TTVEGMKMGG

        50         60         70         80
AFMKIHNDEA KQDFLLGGSS PVADRVEVHT HINDNGVMRM

        90        100        110        120
REVKGGVPLE AKSVTELKPG SYHVMFMGLK KQLKEGDKIP

       130        140        150
VTLKFKNAKA QTVQLEVKTA PMPAMNHGHH HGEAHQH*
```

Figure S1. Sequence of AccA from *N. gonorrhoeae* (UniProt A0AAQ1E0N0), showing the residues in the conserved primary site (green), C-terminal His/Met-rich tail (brown), track (purple), and the leader peptide (underlined).

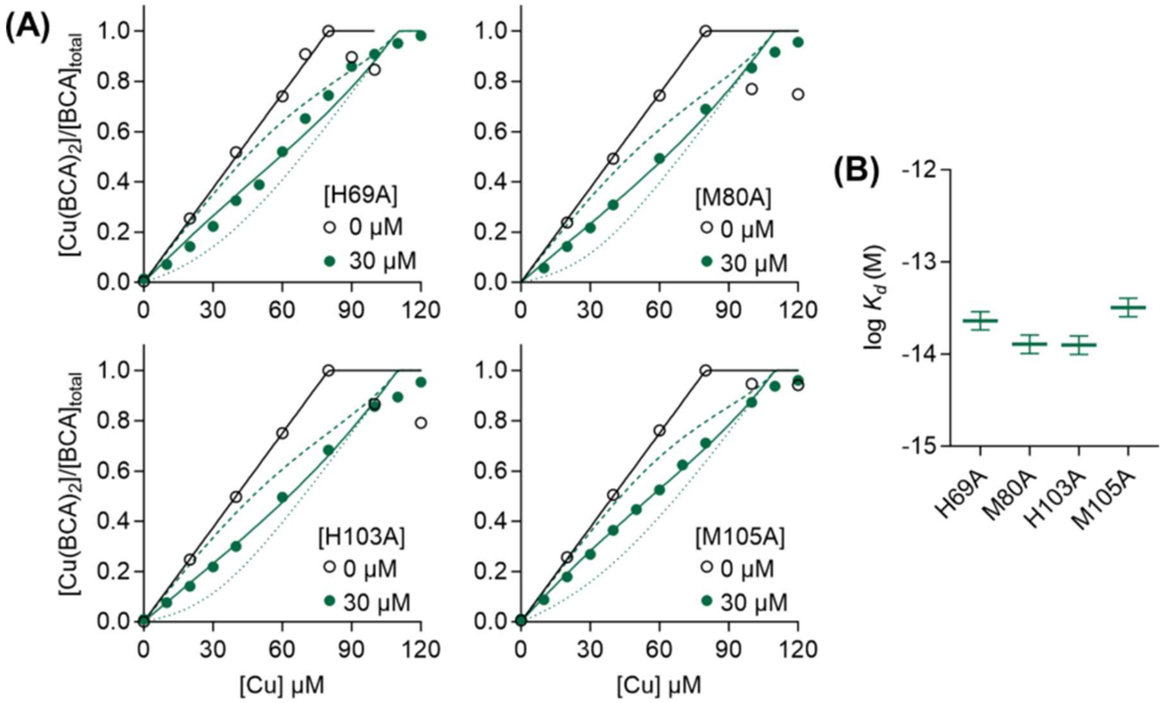

Figure S2. Cu(I)-binding affinities of AccA variants lacking primary site residues. (A) Competition curves between BCA (160 μM) and H69A-AccA, M80A-AccA, H103A-AccA, or M105A-AccA (0 or 30 μM each). Individual data points are shown. Competition curve fits (green solid lines) produced the log K_D values shown in panel G. Control curve fits (black solid lines) and simulated fits for 10X lower (dotted lines) or 10X higher (dashed lines) K_D values are also shown. **(B)** Summary of log K_D values (horizontal lines) obtained from panel (A). Error bars represent ±SEM.

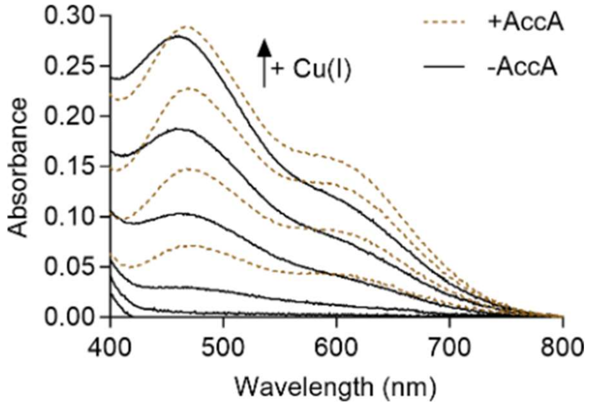

Figure S3. Suspected formation of an AccA-Cu(I)-Fz ternary complex. Changes in the absorbance spectrum of *apo*-Fz (140 μM) upon adding Cu (0 - 70 μM) without (solid lines) or with (dashed lines) AccA (30 μM) in the presence of ascorbate (2 mM).

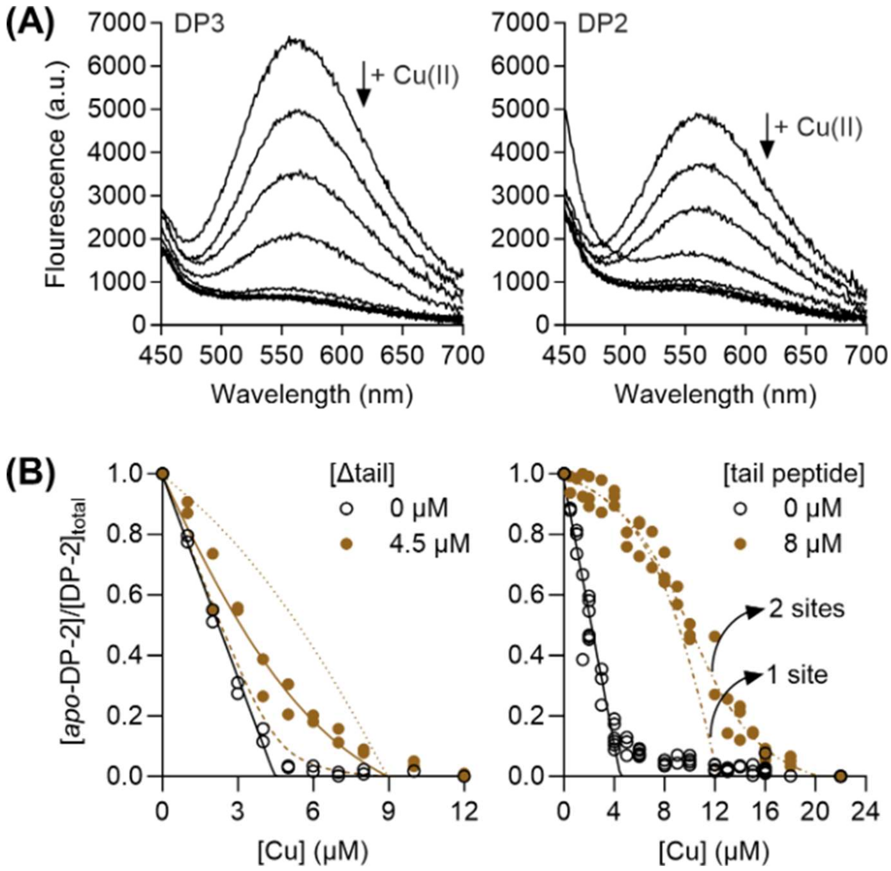

Figure S4. Cu(II)-binding affinities of ∆tail-AccA and the synthetic tail peptide. (A) Changes in the fluorescence emission spectrum of *apo*-DP3 or *apo*-DP2 (4.5 µM each) upon adding Cu (0–10 µM) without ascorbate. **(B)** Competition curves between DP2 (4.5 µM) and the ∆tail-AccA protein or the synthetic tail peptide (0 or 4.5 µM each). The competition approach is shown in Figure 2A in the main text. Individual data points are shown. Competition curve fits (brown solid lines) produced the log K_D values shown in Figure 2G in the main text. Control curve fits (black solid lines), simulated fits for 10X lower (dotted lines) or 10X higher (dashed lines) K_D values, and simulated fits for different values for n (1 or 2; dot-dashed lines) are also shown.

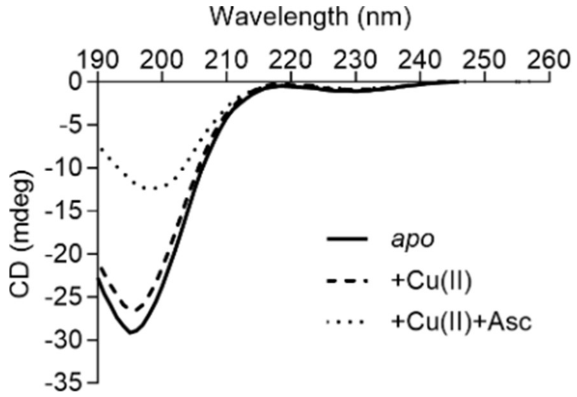

Figure S5. The C-terminal His/Met-rich tail does not adopt a secondary structure. Circular dichroism spectrum of the synthetic tail peptide (50 µM each) in its *apo*-form (solid line), in the presence of 2 eq. Cu(II) without (dashed line) and with (dotted line) excess ascorbate. Each spectrum was collected in 10 mM potassium phosphate buffer (pH 7.4) using the J-1000 Series spectrophotometer with the following parameters: CD scale 200 mdeg/1.0 dOD, scanning speed 50 nm/min, data pitch 1.0 nm, DIT 4 s, bandwidth 3.0 nm, number of accumulations 5.

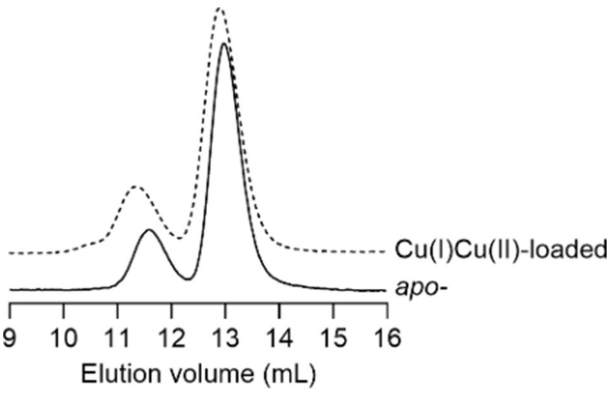

Figure S6. Oligomeric state of AccA in solution. Analytical size exclusion chromatograms of the *apo*- and Cu(I)- and Cu(II)-loaded form of WT-AccA on a calibrated Superdex 75 Increase 10/300 GL column, each showing one main elution peak and retention volume consistent with a monomer.

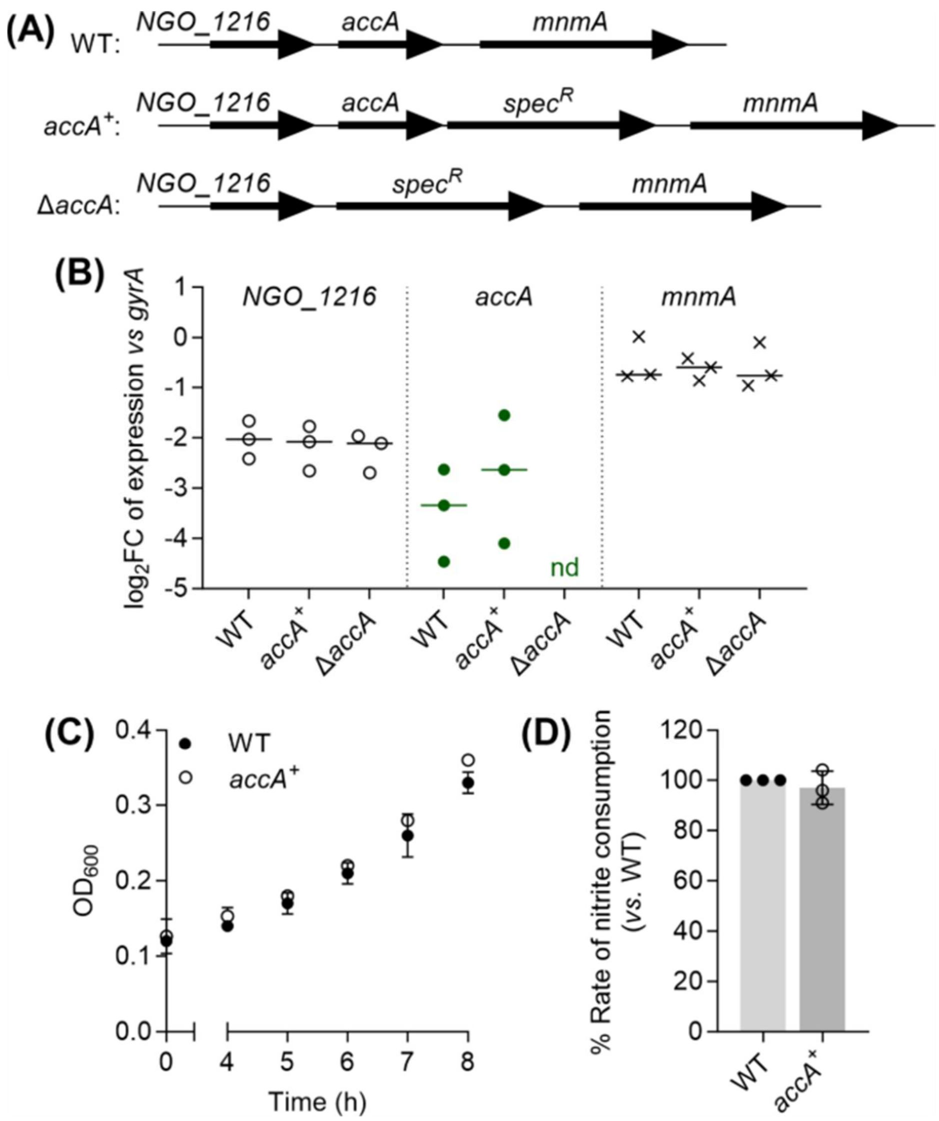

Figure S7. Non-polar insertion of spectinomycin-resistant cassette in *N. gonorrhoeae* mutant strains. (A) Approximate arrangement of genes. **(B)** Expression levels of genes adjacent to the *accA* locus in *N. gonorrhoeae* WT, *accA*+, and Δ*accA* mutant strains. Cells were cultured without added Cu. mRNA levels of each gene were determined by qRT-PCR. Data from individual replicates are shown. Horizontal lines indicate means. nd, not detectable. **(C)** Growth of *N. gonorrhoeae* WT and *accA*+ mutant strains without added Cu. Data points and error bars represent the means (N = 3) and ±SD, respectively. **(D)** Rates of nitrite consumption by whole *N. gonorrhoeae* WT and *accA*+ mutant cells from the 8-hour timepoint in panel C. Data points from individual replicates (N = 3) are shown. Error bars represent ±SD.

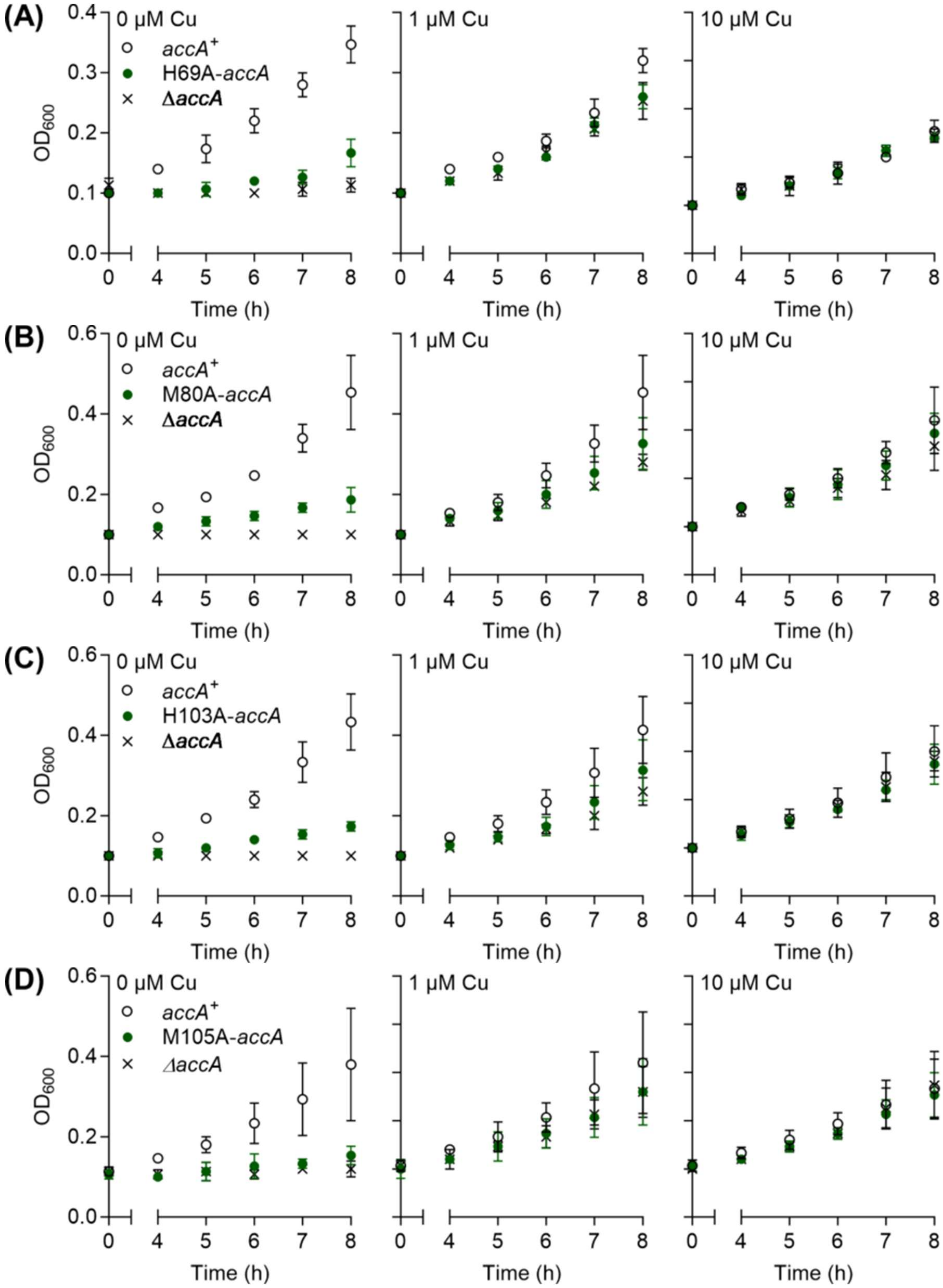
(A)
0 μM Cu
1 μM Cu
10 μM Cu
accA+
H69A-accA
ΔaccA
OD600
Time (h)
(B)
0 μM Cu
1 μM Cu
10 μM Cu
accA+
M80A-accA
ΔaccA
OD600
Time (h)
(C)
0 μM Cu
1 μM Cu
10 μM Cu
accA+
H103A-accA
ΔaccA
OD600
Time (h)
(D)
0 μM Cu
1 μM Cu
10 μM Cu
accA+
M105A-accA
ΔaccA
OD600
Time (h)

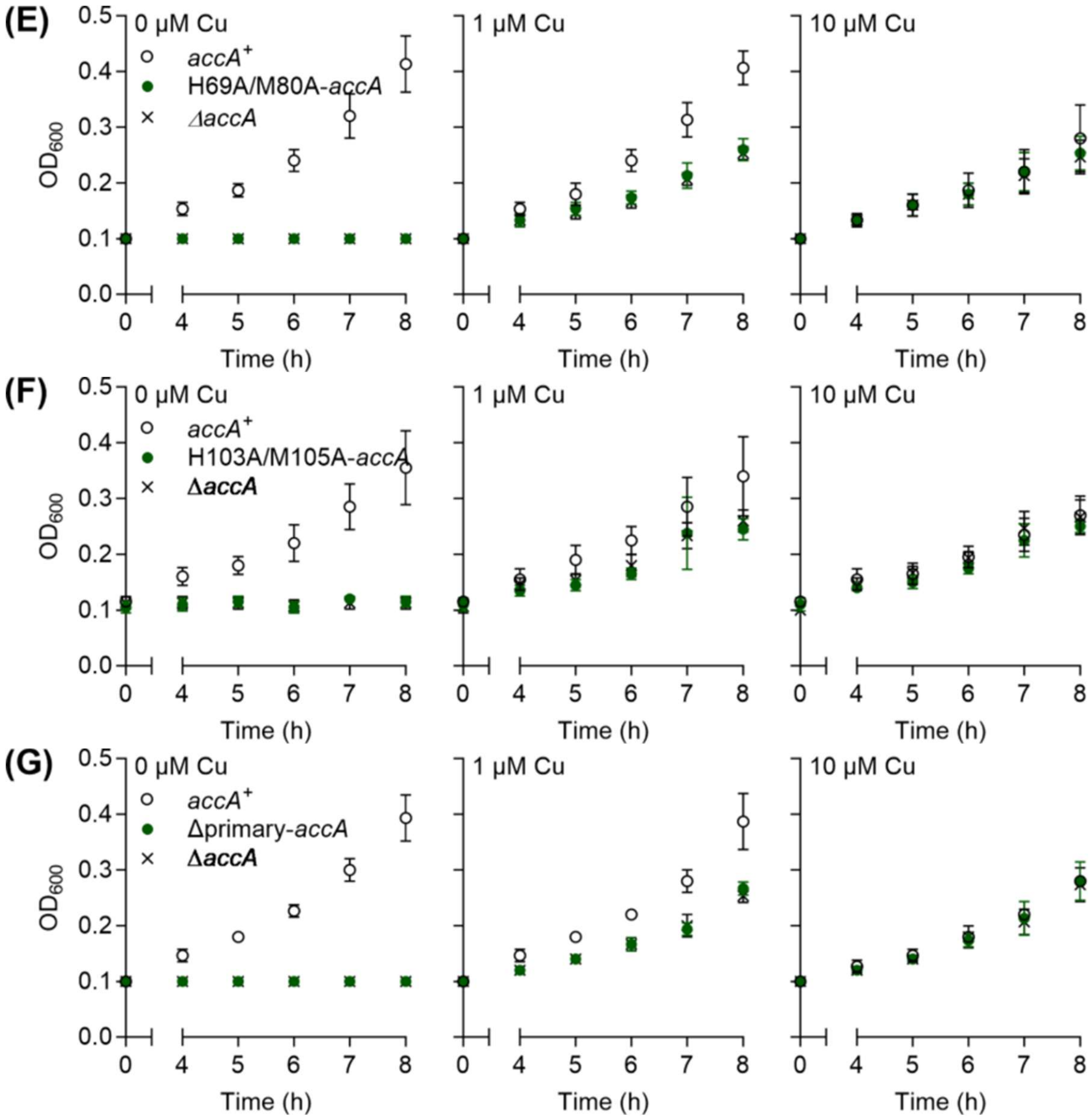

Figure S8. Growth phenotypes of *accA* mutant strains lacking primary site ligands. Growth of *N. gonorrhoeae* **(A)** H69A-*accA,* **(B)** M80A-*accA,* **(C)** H103A-*accA,* **(D)** M105A-*accA,* **(E)** H69A/M80A-*accA,* **(F)** H103A/M105A-*accA,* and **(G)** H69A/M80A/H103A/M105A-*accA* (Δprimary-*accA*) with 0, 1 or 10 μM of added Cu. The *accA*⁺ and Δ*accA* mutant strains, grown under the same conditions as controls, are also shown. Data points and error bars represent means (N = 3) and ±SD, respectively.

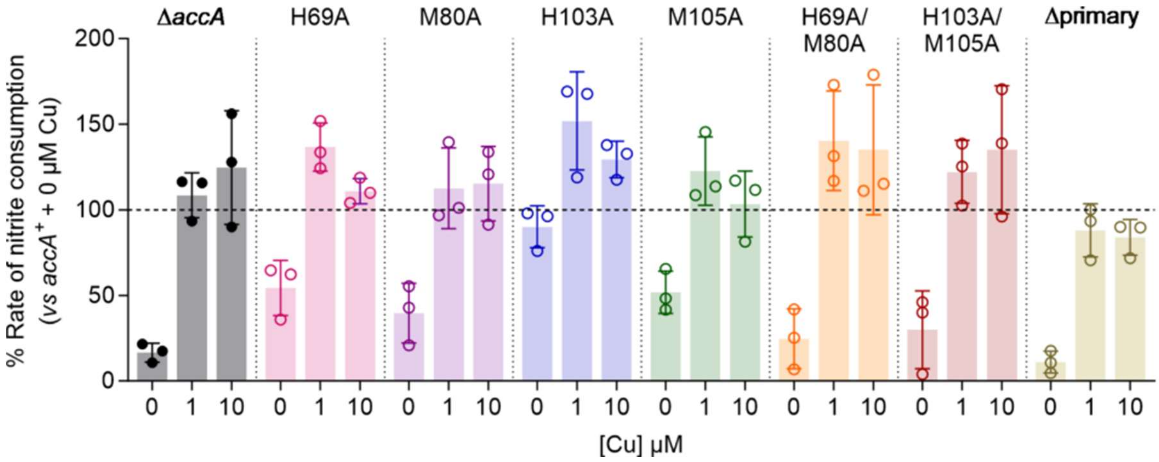

Figure S9. Rates of nitrite consumption by *accA* mutant strains lacking primary site ligands. Cells were cultured for 8 h with and without added Cu. Rates of nitrite consumption by whole cells were normalised to the untreated *accA*$^{+}$ control (dashed horizontal line). The Δ*accA* mutant strain is also shown as a control. Data points, columns, and error bars represent individual replicates (N = 3), means, and ±SD, respectively.

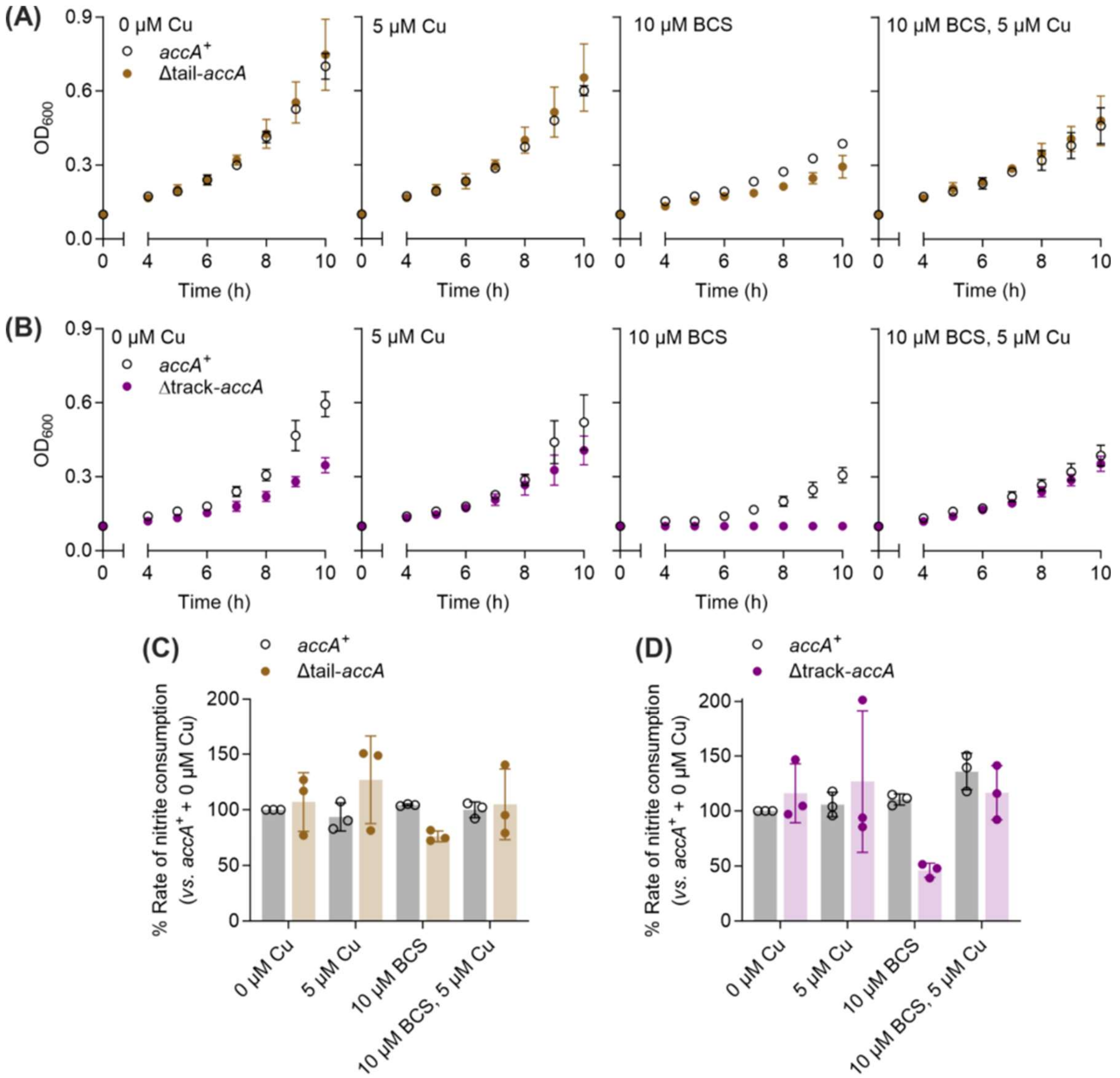

Figure S10. Culture phenotypes of the Δtail-*accA* and Δtrack-*accA* mutant strains. Growth of *N. gonorrhoeae* **(A)** Δtail-*accA* and **(B)** Δtrack-*accA* mutant strains with and without added Cu and/or BCS. Data points and error bars represent means (N = 3) and ±SD, respectively. Growth curves of the *accA*+ control strain under the same conditions are also shown. Rates of nitrite consumption by whole *N. gonorrhoeae* **(C)** Δtail-*accA* and **(D)** Δtrack-*accA* mutant cells from the 8-hour timepoints in panels A and B, respectively. Data points from individual replicates (N = 3) are shown. Error bars represent ±SD.

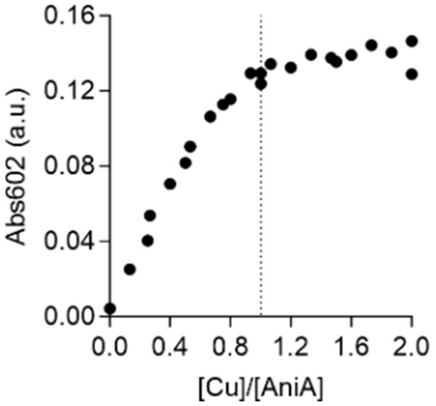

Figure S11. Metalation of the T1Cu site in AniA by Cu(II) ions. Change in the solution absorbance of AniA (70 µM) at 602 nm upon addition of $CuSO_4$ (0–2.0 eq.). Dotted vertical line represents the end point at 1 eq. of Cu. Data from individual replicates (N = 2) are shown.

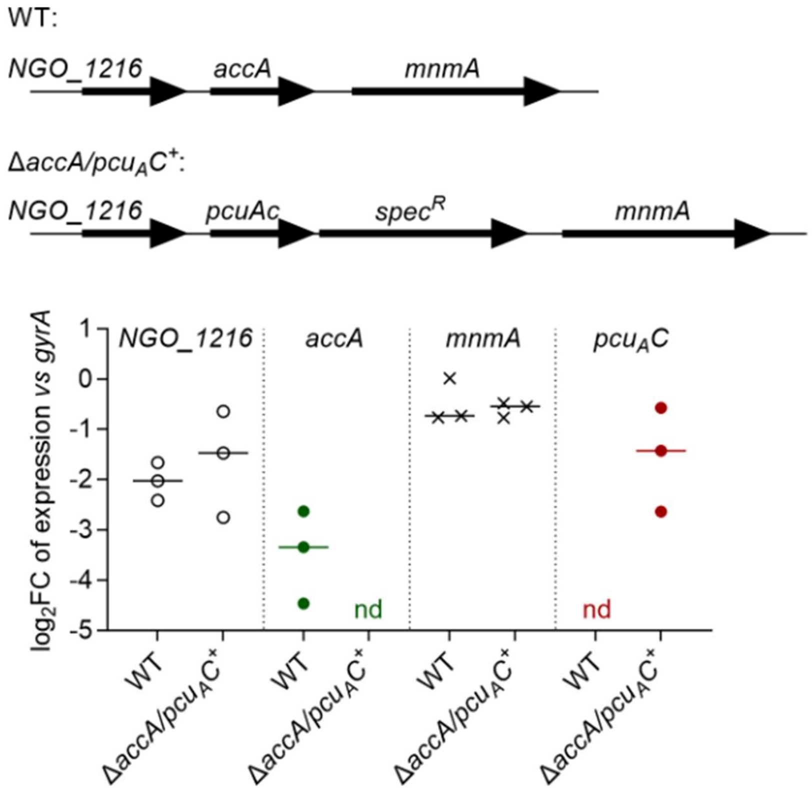

Figure S12. Transcription of *pcu$_A$C* gene in *N. gonorrhoeae*. Expression levels of genes adjacent to the *accA* locus in *N. gonorrhoeae* WT, Δ*accA*, and Δ*accA/pcu$_A$C*$^+$ mutant strains. mRNA levels of each gene were determined by qRT-PCR. Data from individual replicates are shown. Horizontal lines indicate means. nd, not detectable. A schematic diagram showing the approximate arrangements of the genes is shown on top.

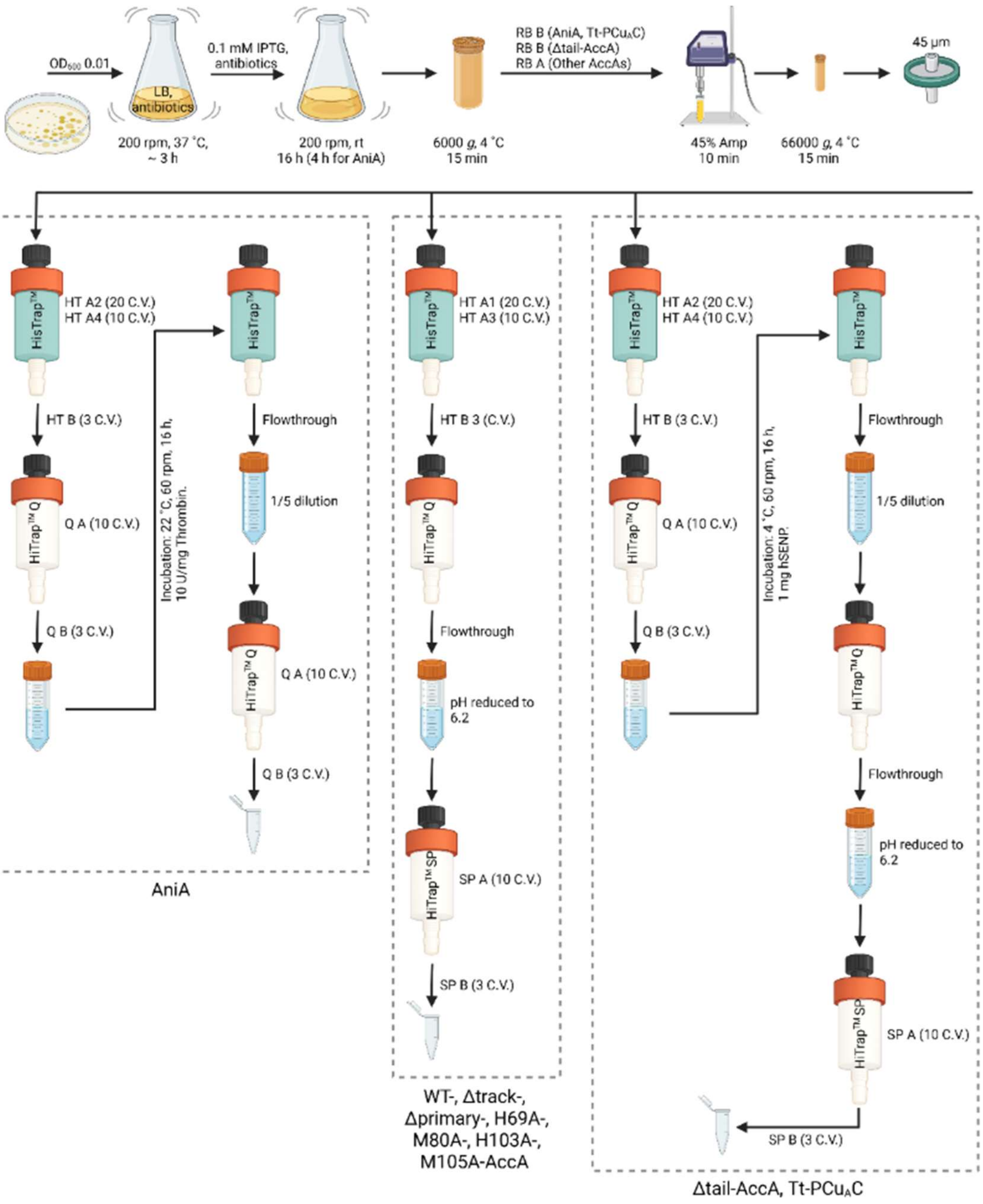

Figure S13. Procedures for overexpression and purification of all proteins in this work. Buffer compositions are in Table S4. Columns are from Cytiva. C.V., column volumes.

Supplementary Tables

Table S1. Bacterial strains used in this study.

Strain	Genetic modification	Description	Antibiotic resistance	Source
N. gonorrhoeae				
1291	WT	Wild-type parent strain	-	W. Shafer (Emory University, USA)
1291	*accA*+	As WT strain but with a promoterless *specR* cassette added between the *accA* stop codon and transcriptional terminator	Spectinomycin	This work
1291	Δ*accA*	As *accA*+ strain but the entire *accA* gene deleted	Spectinomycin	This work
1291	H69A-*accA*	As *accA*+ strain but the His69 codon mutated to encode Ala	Spectinomycin	This work
1291	M80A-*accA*	As *accA*+ strain but the Met80 codon mutated to encode Ala	Spectinomycin	This work
1291	H103A-*accA*	As *accA*+ strain but the His103 codon mutated to encode Ala	Spectinomycin	This work
1291	M105A-*accA*	As *accA*+ strain but the Met105 codon was mutated to encode Ala	Spectinomycin	This work
1291	H69A/M80A-*accA*	As *accA*+ strain but the His69 and Met80 codons were mutated to encode Ala	Spectinomycin	This work
1291	H103A/M105A-*accA*	As *accA*+ strain but the His103 and Met105 codons were mutated to encode Ala	Spectinomycin	This work
1291	Δprimary-*accA*	As *accA*+ strain but the His69, Met80, His103 and Met105 codons were mutated to encode Ala	Spectinomycin	This work
1291	Δtrack-*accA*	As *accA*+ strain but the Met36, His71, Met78 and Met107 codons were mutated to encode Ala	Spectinomycin	This work
1291	Δtail-*accA*	As *accA*+ strain but bases 423 to 471 encoding the C-terminal "tail" in the *accA* gene were deleted	Spectinomycin	This work
1291	Δ*accA*/pcuAC+	As *accA*+ strain but with gene encoding mature Tt-PCuAC replacing the gene encoding mature AccA. The AccA signal peptide is retained.	Spectinomycin	This work

Escherichia coli

DH5α	-	Standard cloning strain	-	Lab collection
BL21(DE3) pLysS	-	Expression host with rare codons	Chloramphenicol	Lab collection
BL21 (DE3) Rosetta 2	-	Expression host with rare codons	Chloramphenicol	Lab collection
BL21(DE3) pLysS	pET11b::*accA*	Overexpression strain for wild-type AccA	Chloramphenicol, Ampicillin	This work
BL21(DE3) pLysS	pET11b::H69A-*accA*	Overexpression strain for H69A-AccA variant	Chloramphenicol, Ampicillin	This work
BL21(DE3) pLysS	pET11b::M80A-*accA*	Overexpression strain for M80A-AccA variant	Chloramphenicol, Ampicillin	This work
BL21(DE3) pLysS	pET11b::H103A-*accA*	Overexpression strain for H103A-AccA variant	Chloramphenicol, Ampicillin	This work
BL21(DE3) pLysS	pET11b::M105A-*accA*	Overexpression strain for M105A-AccA variant	Chloramphenicol, Ampicillin	This work
BL21 (DE3) Rosetta 2	pET11b::Δprimary-*accA*	Overexpression strain for H69A/M80A/H103A/M105A-AccA variant	Chloramphenicol, Ampicillin	This work
BL21 (DE3) Rosetta 2	pET11b::Δtrack-*accA*	Overexpression strain for M36A/H71A/M78A/M107A-AccA variant	Chloramphenicol, Ampicillin	This work
BL21 (DE3) Rosetta 2	pSATL::Δtail-*accA*	Overexpression strain for AccA variant missing M142 to H157, carrying an N-terminal, cleavable His_6-SUMO tag.	Chloramphenicol, Ampicillin	This work
BL21 (DE3) CodonPlus(+)	pSATL::Tt-PCuAC	Overexpression strain for the soluble domain of Tt-PCuAC fused to the AccA N-terminal leader sequence, carrying an N-terminal, cleavable His6-SUMO tag	Chloramphenicol, Ampicillin	This work
BL21 (DE3) Rosetta 2	pET29a::*aniA*	Overexpression strain for the soluble domain of AniA from Ala24 to Ala364	Chloramphenicol, Kanamycin	This work

Table S2. Vectors and plasmids used in this study.

Plasmid name	Description	Antibiotic resistance	Source
**Empty vectors**			
pCTS32	Source of *SpecR* cassette	Spectinomycin	M. Apicella (Iowa)
pET-11b	Overexpression vector	Ampicillin	Lab collection
pSATL	Overexpression vector with an N-terminal, cleavable His_6-SUMO tag	Ampicillin	T. Blower (Durham)
pTRB479	pUC19 plasmid, after removal of the native *Bsa*I site	Ampicillin	T. Blower (Durham)
pUC19	Cloning vector	Ampicillin	Lab collection
pET-29a	Overexpression vector with a C-terminal 6xHis-tag	Kanamycin	N. Robinson (Durham)
**For making *N. gonorrhoeae* mutant strains**			
pUC19::*accA-specR*	Plasmid containing wild-type *accA* gene	Ampicillin, Spectinomycin	This work
pUC19::H69A-*accA*	Plasmid containing H69A-*accA* mutant gene	Ampicillin, Spectinomycin	This work
pUC19::M80A-*accA*	Plasmid containing M80A-*accA* mutant gene	Ampicillin, Spectinomycin	This work
pUC19::H103A-*accA*	Plasmid containing H103A-*accA* mutant gene	Ampicillin, Spectinomycin	This work
pUC19::M105A-*accA*	Plasmid containing M105A-*accA* mutant gene	Ampicillin, Spectinomycin	This work
pUC19::H69A/M80A-*accA*	Plasmid containing H69A/M80A-*accA* mutant gene	Ampicillin, Spectinomycin	This work
pUC19::H103A/M105-*accA*	Plasmid containing H103A/M105A-*accA* mutant gene	Ampicillin, Spectinomycin	This work
pUC19::Δprimary-*accA*	Plasmid containing H69A/M80A/H103A/M105A-*accA* mutant gene	Ampicillin, Spectinomycin	This work
pUC19::Δtrack-*accA*	Plasmid containing M36A/H71A/M78A/M107A-*accA* mutant gene	Ampicillin, Spectinomycin	This work
pUC19::Δtail-*accA*	Plamsid containing accA mutant gene missing amino acids M142 to H157	Ampicillin, Spectinomycin	This work
pUC19::*pcuAC-specR*	Plamsid with the gene encoding mature domain of Tt-PCuAC replacing the gene encoding mature domain of AccA. The AccA leader sequence is retained.	Ampicillin, Spectinomycin	This work

For protein overexpression

pET-11b::*accA*	Plasmid for overexpression of wild-type AccA	Ampicillin	This work
pET-11b::H69A-*accA*	Plasmid for overexpression of H69A-AccA variant	Ampicillin	This work
pET11b::M80A-*accA*	Plasmid for overexpression of M80A-AccA variant	Ampicillin	This work
pET11b::H103A-*accA*	Plasmid for overexpression of H103A-AccA variant	Ampicillin	This work
pET11b::M105A-*accA*	Plasmid for overexpression of M105A-AccA variant	Ampicillin	This work
pET11b::Δprimary-*accA*	Plasmid for overexpression of H69A/M80A/H103A/M105A-AccA variant	Ampicillin	This work
pET11b::Δtrack-*accA*	Plasmid for overexpression of M36A/H71A/M78A/M107A-AccA variant	Ampicillin	This work
pSATL::Δtail-*accA*	Plasmid for overexpression of AccA variant missing M142 to H157, carrying an N-terminal, cleavable His_6-SUMO tag	Ampicillin	This work
pSATL::Tt-PCuAC	Plasmid for overexpression of the soluble domain of Tt-PCuAC fused to the AccA N-terminal leader sequence, carrying an N-terminal, cleavable His6-SUMO tag	Ampicillin	This work
pET29a::*aniA*	Plasmid for overexpression of wild-type AniA	Kanamycin	This work

Table S3. Primers used in this study. All primers were synthesised commercially (Integrated DNA Technologies).

Name of construct, target gene, or target protein	Primer name	Sequence (5'-->3')	PCR template
For constructing Golden Gate-assembled constructs			
accA-spec	5' upstream-F	CAGTTGGGTCTCCGGAGGACAAACGCATCTTGATTATCG	*N. gonorrhoeae* 1291 gDNA
	5' upstream-R	CAGTTGGGTCTCCATCTTCCTGCTCCTTTAATATCAG	
	accA-F	CAGTTGGGTCTCCAGATGAAAAAATTATTGGCAGCCG	
	accA-R	CAGTTGGGTCTCCTTAGTGCTGATGCGCTTC	
	3' downstream-F	CAGTTGGGTCTCGTTTCTGCTGGAAATATTTGAAATGC	
	3' downstream-R	CAGTTGGGTCTCGATGGCGTGAAACTCAAATCGTTCA	
	specR-F	CAGTTGGGTCTCCCTAAAATAGGTACTAATGAAAATAGTGAGG	pCTS32
	specR-R	CAGTTGGGTCTCCGAAAGGTGTTTCCACCATTTTT	
	accA GG-F	GCATTTTTTTGGGTTTCCGAAA	*accA-spec* Golden Gate assembly mixture
	accA GG-R	CAAACCTTTGAGCAGGTAATG	
Δ*accA-spec*	Δ*accA*-F	AATAGGTACTAATGAAAATAGTGAGG	pUC19::*accA-spec*
	Δ*accA*-R	CTTCCTGCTCCTTTAATATC	
Splice overlap extension PCR external	SOE-F	GCTGCAAGGCGATTAAGTTGGGTAACGC	pUC19::*accA-spec* (wild-type or variant)
	SOE-R	GAGGAAGCGGAAGAGCGCCCAATAC	
H69A-*accA*	H69A SOE-F	GCGTCGAAGTGGCAACCCACATCAAC	pUC19::*accA-spec*
	H69A SOE-R	GTTGATGTGGGTTGCCACTTCGACGC	
M105A-*accA*	M105A SOE-F	CAGCTATCACGTGGCATTTATGGGTTTGA	
	M105A SOE-R	TCAAACCCATAAATGCCACGTGATAGCTG	
H103A/M105A-*accA*	M103A/M105A SOE-F	ACCCGGCAGCTATGCAGTGGCATTTATGGGTTTGA	
	H103A/M105A SOE-R	TCAAACCCATAAATGCCACTGCATAGCTGCCGGGT	

ΔCu tail-*accA*	ΔC-terminal SOE-F	AACCGCGCCGTAAAATAGGTACTAATGAAAATAGTGAGGAG GATATATTTGAATACATAC	
	ΔC-terminal SOE-R	ACCTATTTTACGGCGCGGTTTTGACTTCCA	
Δ*accA/pcuAC*+	accA-F	CAGTTGGGTCTCCAGATGAAAAAATTATTGGCAGCCG	commercially synthesised pcuAC gene
	pcuAC-R	GGCTACGGTCTCTAGTACCTATTTCAGCGTGCTTCAACCGG	
<u>For transformation of Golden Gate-assembled constructs into *N. gonorrhoeae* 1291</u>			
accA-spec (wild-type or variant)	linearisation-F	GAGCAGATTGTACTGAGAGTGC	All Golden Gate assembled constructs
	linearisation-R	GAGCGCAGCGAGTCAGTGAG	
<u>For protein overexpression</u>			
AccA (wild-type or variant)	AccA O/E-F	CATGAGCATATGAAAAAATTATTGGCAGCCGTG	pUC19::*accA-spec* (wild-type or variant)
	AccA O/E-R	CATGAGGGATCCTTAGTGCTGATGCGC	
AniA	AniA O/E-F	CAGCAGCATATGGCCGCACAAGCTACC	*N. gonorrhoeae* 1291 gDNA
	AniA O/R	CATCTCGAGGCTGCCGCGCGGTACGGCGT AAGCGGTATC	

Tt-PCuAC	PCuAC O/E-F	CAACAGCAGACGGGAGGTCAAGTTGTACGTGAAGGGTGG	Golden Gate assembled construct
	PCuAC O/E-R	GCGAGAACCAAGGAAAGGTTATTAGCGTGCTTCAACCGGCAAAAC	
For qPCR analyses			
gyrA	gyrA-qPCR-F	TCCACCGATCCGAAGTTGC	cDNA from *N. gonorrhoeae* 1291 RNA
	gyrA-qPCR-R	CGCAGTTTACGACACCATCG	
mnmA	mnmA-qPCR-F	GTCGTTGTCGTCGTTTTCCC	
	mnmA-qPCR-R	GTCGATTCTTCCGTAACCGC	
NGO_1216	1216-qPCR-F	ATAATCAACGGCAGGCGGAC	
	1216-qPCR-R	ATTCCGTCAGGTTTTGTGGC	
accA	accA-qPCR-F	CCTTTGACTTCGCGCATAC	
	accA-qPCR-F	ACTTTTTGCTCGTCGGAAGC	
pcuAC	pcuAC-qPCR-F	CTTTGCGCTTAGTTGGGG	
	pcuAC-qPCR-R	GGGCGCATTCCCATTAC	

Table S4. Compositions of all buffers used for purification of proteins in this study.

Buffer	Composition
Resuspension buffer (RB) A	50 mM Tris-HCl pH 8.0, 150 mM NaCl, 5 mM imidazole, 15 w/v % glycerol
Resuspension buffer (RB) B	50 mM Tris-HCl pH 8.0, 150 mM NaCl, 25 mM imidazole, 15 w/v % glycerol
His-Trap (HT) A1	50 mM Tris-HCl pH 8.0, 150 mM NaCl, 5 mM imidazole
His-Trap (HT) A2	50 mM Tris-HCl pH 8.0, 150 mM NaCl, 25 mM imidazole
His-Trap (HT) A3	50 mM Tris-HCl pH 8.0, 5 mM imidazole
His-Trap (HT) A4	50 mM Tris-HCl pH 8.0, 25 mM imidazole
His-Trap (HT) B	50 mM Tris-HCl pH 8.0, 150 mM NaCl, 250 mM imidazole
Q-Column (Q) A	50 mM Tris-HCl pH 8.0
Q-Column (Q) B	50 mM Tris-HCl pH 8.0, 250 mM NaCl
SP-Column (SP) A	50 mM MOPs pH 6.5
SP-Column (SP) B	50 mM MOPs pH 7.2, 150 mM NaCl, 15 w/v % glycerol

Table S5. Theoretical and actual masses of all proteins used in this study.

	Mass (Da)	
**Protein**	**Theoretical**	**Actual**
WT AccA	15166	15166
H69A-AccA	15100	15100
M80A-AccA	15106	15106
H103A-AccA	15100	15100
M105A-AccA	15106	15106
∆Cu primary-AccA	14914	14914
∆Cu track-AccA	14920	14920
∆Cu tail-AccA	13356	13356
Tt-PCuAC	13294	13295
AniA	34789	34788

Table S6. Properties of Cu(I)- and Cu(II)-binding competitor probes used in this study.

Probe	Cu complex	β_2 (M^{-2})	λ_{max} (nm) for holo-complex	ε (cm^{-1} M^{-1})
BCS	$[Cu^{I}(BCS)_2]^{3-}$	6.3×10^{19}	483	13000
BCA	$[Cu^{I}(BCA)_2]^{3-}$	1.6×10^{17}	562	7900
Fz	$[Cu^{I}(Fz)_2]^{3-}$	1.3×10^{15}	470	4320

			F (nm) for *apo*-probe	
**Probe**	**Cu complex**	***KD*** **(M)**	$F_{(em)}$	$F_{(ex)}$
DP-2	Cu^{II}(DP2)	7.94E-11	350	550
DP-3	Cu^{II}(DP3)	5.01E-13	350	550

Table S7. Crystallographic data collection and refinement statistics.

PDB deposition ID	9YAH
**Data collection**	
Wavelength	1.377
Resolution range (Å)	44.97-2.90
Space group	$P3_221$
**Unit cell parameters**	
a, b, c (Å)	103.863 103.863 31.609
a,b,g (^{0})	90 90 120
Total reflections	87608
Unique reflections	8469
Multiplicity	10.3(9.2)
Completeness (%)	99.34 (96.14)
Mean I/sigma(I)	12.19(2.84)
Wilson B-factor	77.79
R-merge[a]	0.132(0.713)
R-meas[b]	0.139 (0.7551)
CC½[c]	0.994(0.854)
**Refinement**	
Resolution	44.97-2.90 (3.08-2.90)
R-factor/R-free[d]	0.22/0.27 (0.35/0.38)
**Number of non-hydrogen atoms**	
Macromolecules	930
Ligands	8
Solvent	0
Protein residues	121
RMS (bonds) (Å)	0.003
RMS (angles) (^{0})	0.599
Rotamer outliers (%)	0
Clash score[e]	14.2
**Ramachandran statistics**	
Favored (%)	94.12
Allowed (%)	5.88
Outliers (%)	0
**Average B-factor (Å^2)**	
All atoms	75.73
Macromolecules	75.7
Ligands	78.64

Values in parentheses are for the outer resolution shell.

[a] *R*merge = ∑*hkl* ∑*i* |*Ii(hkl)* - <*I(hkl)*>|/ ∑*hkl* ∑*iIi(hkl)*.

[b] *R*meas = ∑*hkl* [*N*/(*N* - 1)]1/2 × ∑*i* |*Ii(hkl)* - <*I(hkl)*>|/ ∑*hkl* ∑*iIi(hkl)*, where *Ii(hkl)* is the *i*th observation of reflection *hkl*, <*I(hkl)*> is the weighted average intensity for all observations *i* of reflection *hkl* and *N* is the number of observations of reflection *hkl*.

[c] *CC½* is the correlation coefficient between symmetry equivalent intensities from random halves of the dataset.

[d] The data set was split into "working" and "free" sets consisting of 95 and 5% of the data,
respectively. The free set was not used for refinement. The R-factors *R*work and *R*free are calculated using the respective sets of data as follows: *R* = ∑(| *F*obs - *F*calc |)/∑| *F*obs |, where *F*obs and *F*calc are the observed and calculated structure factor amplitudes, respectively.

[e] Calculated using MolProbity(10).

Dataset S1 (separate file). DynaFit scripts used in this work.

Supplementary References

1. Xiao Z, Brose J, Schimo S, Ackland SM, La Fontaine S, Wedd AG. Unification of the Copper(I) Binding Affinities of the Metallo-chaperones Atx1, Atox1, and Related Proteins. J Biol Chem. 2011;286(13):11047–55.

2. Steichen CT, Shao JQ, Ketterer MR, Apicella MA. Gonococcal Cervicitis: A Role for Biofilm in Pathogenesis. J Infect Dis. 2008;198(12):1856–61.

3. Dillard JP. Genetic Manipulation of Neisseria gonorrhoeae. Curr Protoc Microbiol. 2011;0 4:Unit4A.2.

4. Boulanger MJ, Murphy MEP. Crystal structure of the soluble domain of the major anaerobically induced outer membrane protein (AniA) from pathogenic *Neisseria*: a new class of copper-containing nitrite reductases1. J Mol Biol. 2002;315(5):1111–27.

5. Barreiro DS, Oliveira RNS, Pauleta SR. Biochemical Characterization of the Copper Nitrite Reductase from Neisseria gonorrhoeae. Biomolecules. 2023;13(8):1215.

6. Sikora AE, Mills RH, Weber JV, Hamza A, Passow BW, Romaine A, et al. Peptide Inhibitors Targeting the Neisseria gonorrhoeae Pivotal Anaerobic Respiration Factor AniA. Antimicrob Agents Chemother. 2017;61(8):10.1128/aac.00186-17.

7. Ruijter JM, Ramakers C, Hoogaars WMH, Karlen Y, Bakker O, van den Hoff MJB, et al. Amplification efficiency: linking baseline and bias in the analysis of quantitative PCR data. Nucleic Acids Res. 2009;37(6):e45.

8. Williams CJ, Headd JJ, Moriarty NW, Prisant MG, Videau LL, Deis LN, et al. MolProbity: More and better reference data for improved all-atom structure validation. Protein Sci. 2018;27(1):293-315
